# Structural studies of the shortest extended synaptotagmin with only two C2 domains from *Trypanosoma brucei*

**DOI:** 10.1016/j.isci.2021.102422

**Published:** 2021-04-20

**Authors:** Emma Stepinac, Nicolas Landrein, Daria Skwarzyńska, Patrycja Wójcik, Johannes Lesigang, Iva Lučić, Cynthia Y. He, Mélanie Bonhivers, Derrick R. Robinson, Gang Dong

**Affiliations:** 1 Max Perutz Labs, Vienna Biocenter, Center for Medical Biochemistry, Medical University of Vienna, 1030 Vienna, Austria; 2 University of Bordeaux, CNRS, Microbiologie Fondamentale et Pathogénicité, UMR 5234, 33000 Bordeaux, France; 3 Silesian University of Technology, Gliwice, Poland; 4 Department of Biological Sciences, Center for BioImaging Sciences, National University of Singapore, Singapore, Singapore

**Keywords:** Biochemistry, Medical Biochemistry, Biochemistry Applications

## Abstract

Extended synaptotagmins (E-Syts) localize at membrane contact sites between the endoplasmic reticulum (ER) and the plasma membrane to mediate inter-membrane lipid transfer and control plasma membrane lipid homeostasis. All known E-Syts contain an N-terminal transmembrane (TM) hairpin, a central synaptotagmin-like mitochondrial lipid-binding protein (SMP) domain, and three or five C2 domains at their C termini. Here we report an uncharacterized E-Syt from the protist parasite *Trypanosoma brucei*, namely, TbE-Syt. TbE-Syt contains only two C2 domains (C2A and C2B), making it the shortest E-Syt known by now. We determined a 1.5-Å-resolution crystal structure of TbE-Syt-C2B and revealed that it binds lipids via both Ca^2+^- and PI(4,5)P_2_-dependent means. In contrast, TbE-Syt-C2A lacks the Ca^2+^-binding site but may still interact with lipids via a basic surface patch. Our studies suggest a mechanism for how TbE-Syt tethers the ER membrane tightly to the plasma membrane to transfer lipids between the two organelles.

## Highlights

• We identified a new type of extended synaptotagmin (E-Syt) in *Trypanosoma brucei*
• TbE-Syt is the shortest known E-Syt with only two C2 domains, C2A and C2B
• TbE-Syt-C2B binds lipids via both Ca^2+^- and PI(4,5)P_2_-dependent means
• Unlike all other known E-Syts, TbE-Syt-C2A and C2B are connected by a flexible loop

## Graphical abstract

**Figure:**
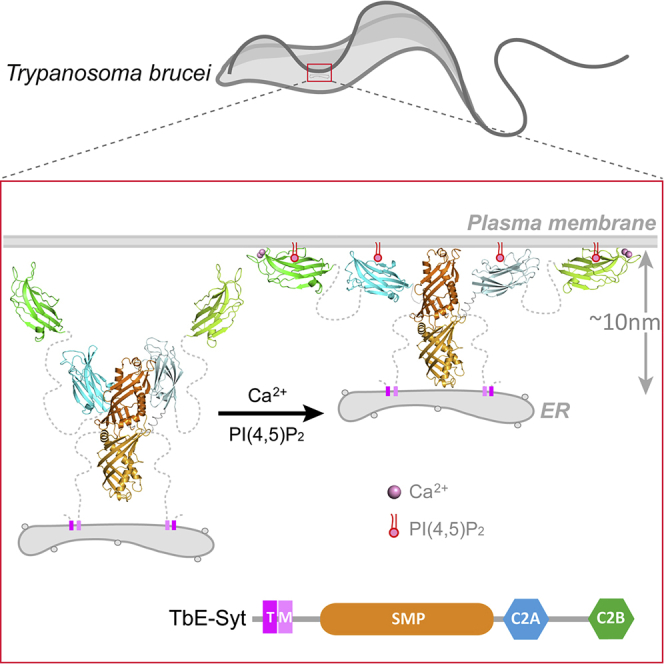

## Introduction

Eukaryotic cells are compartmentalized into membrane-bound organelles, which are defined by their unique membrane lipid composition and specific functions. Membrane trafficking acts to transfer materials between organelles over long distances, whereas closely apposed membranes from two organelles can form membrane contact sites (MCSs) to exchange lipids between them without undergoing membrane fusion (Hayashi et al., 2008; Lahiri et al., 2015; Levine and Loewen, 2006). Extended synaptotagmins (E-Syts) are a family of evolutionarily conserved proteins localizing at the MCS between the endoplasmic reticulum (ER) and the plasma membrane and mediate lipid transfer between them (Fernandez-Busnadiego et al., 2015; Giordano et al., 2013). E-Syts belong to the tubular lipid binding (TULIP) superfamily. Proteins from this group contain hydrophobic tunnels in their TULIP domains and often function in lipid traffic and signaling by carrying lipids through the aqueous phase of the cell (Beamer et al., 1997; Kopec et al., 2011; Qiu et al., 2007; Wong and Levine, 2017). The name of E-Syt derives from synaptotagmin (Syt), which is a protein anchored on the membrane of secretory vesicles and acting as a tether between the vesicles and their target sites during exocytosis (Brose et al., 1992; Elferink et al., 1993; Perin et al., 1991). However, E-Stys differ from Syts in both their localization and function.

E-Syts are present in plants, fungi, and animals (Craxton, 2007; Giordano et al., 2013; Toulmay and Prinz, 2012). There are three E-Syts, E-Syt1, 2 and 3, in mammals (Min et al., 2007). These E-Syts all have two tandem hydrophobic helices at their N terminus, which form a transmembrane (TM) hairpin anchoring them within the ER membrane (Giordano et al., 2013). The E-Syts also contain a central SMP (synaptotagmin-like mitochondrial-lipid binding protein) domain and five (E-Syt1) or three (E-Syt2 and 3) C2 domains at their C terminus (Min et al., 2007). The first two C2 domains, C2A and C2B, of human E-Syt2 (HsE-Syt2) form a rigid V-shaped conformation, with only C2A capable of binding Ca^2+^ (Xu et al., 2014). HsE-Syt2 also forms a homodimer via its SMP domain, which generates a hydrophobic tunnel along the dimer for lipid binding and transfer between the ER and the plasma membrane (Schauder et al., 2014).

Trypanosomes are kinetoplastids that are thought to be one of the earliest branching lineages of eukaryotes (Simpson et al., 2006). Consequently, they are among the most divergent eukaryotes on the planet. Studies on conserved proteins in trypanosomes could thus help us understand the fundamental function and evolution of these proteins. Here we report our structural and functional characterization of a unique, single-copy E-Syt from *Trypanosoma brucei*.

Similar to all mammalian E-Syts, *T. brucei* E-Syt (TbE-Syt, Tb927.10.13740) is predicted to also have an N-terminal TM hairpin and a central SMP domain, which is a defining element of the E-Syt protein family. However, TbE-Syt contains only two C2 domains, which we named C2A and C2B based on their topological and structural similarities to their counterparts in mammalian E-Syts. We determined a 1.5-Å-resolution crystal structure of TbE-Syt-C2B, which revealed that it adopts a type II topology and has two tightly bound Ca^2+^ ions. These Ca^2+^ ions are coordinated by a few negatively charged residues from two extended loops. Additionally, the structure shows an extensive surface patch consisting of mostly basic residues. Our *in vitro* liposome-binding data demonstrated that TbE-Syt-C2B bound lipids via both Ca^2+^-dependent and phosphatidylinositol 4,5-bisphosphate [PI(4,5)P_2_]-dependent means through these two sites. Homology modeling analyses showed that TbE-Syt-C2A lacks Ca^2+^-binding sites, which is distinct from the Ca^2+^-binding C2A in all mammalian E-Syts. However, TbE-Syt-C2A also contains a similar basic patch to that on TbE-Syt-C2B. Furthermore, our analyses suggest that TbE-Syt-C2A and C2B are connected by a flexible linker, which is also different from the rigid and compact V-shaped conformation of C2A-C2B in mammalian E-Syts. TbE-Syt localized to a specialized ER subdomain in *T. brucei*, and these distinct features of TbE-Syt may correlate with its functions there.

## Results

### TbE-Syt localizes to the ER via its N-terminal transmembrane hairpin

**Figure 1:**
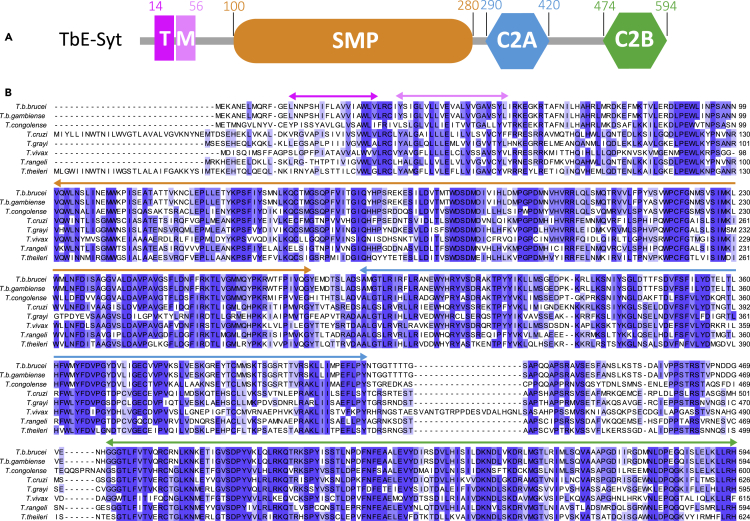
Domain arrangement and conservation analyses of TbE-Syt (A) Schematic illustration of the predicted domains in TbE-Syt. Amino acids in primary structure are shown above the schematic. (B) Sequence alignments of TbE-Syt homologs from various trypanosome species. Boundaries of the predicted domains are marked by arrows above the sequences with the same color schemes as in (A). The alignments were carried out using the option of “Muscle with defaults” in Jalview, with shading indicating the degree of conservation.

The gene Tb927.10.13740 from *T. brucei* encodes a protein of 594 residues and was previously annotated as a putative synaptotagmin (Aslett et al., 2010). Our preliminary bioinformatics analyses suggested that this protein contains two TM motifs (aa14−31 and 34–56), an SMP domain (aa100-280), and two C2 domains (C2A: aa290−420; C2B: aa474−594) (Figure 1A). Sequence alignment of homologs from various trypanosome species showed that all these domains, except for the first TM motif, are highly conserved, whereas the short N-terminal extension and the connecting loop between the two C2 domains are both divergent and variable in length (Figure 1B). Based on its similar overall domain organization to mammalian E-Syts and characteristic localization to the cortical ER revealed by our studies, we provisionally renamed this protein *T. brucei* E-Syt (TbE-Syt).

To assess the localization of TbE-Syt in *T. brucei*, we generated a cell line which inducibly overexpressed TbE-Syt-myc and domains and analyzed by western blotting (WB) the whole cell (WC) or the cytoskeleton-associated fraction of detergent-extracted cells (CSK). The expression level was different among the constructs preventing further WB quantitative comparison (Figures S1A and S1B). However, the analysis showed that both TbE-Syt (FL) and TbE-Syt deleted of the C2B domain (T1) co-purified with the CSK fraction, whereas both the C2B domain (T2) and the TM domain (T3) alone were only found in WC suggesting no or poor CSK association. Nevertheless, the TM domain is required for CSK association as TbE-Syt deleted of its TM (T4) or deleted of both TM and C2B domains (T5) were all soluble.

**Figure 2:**
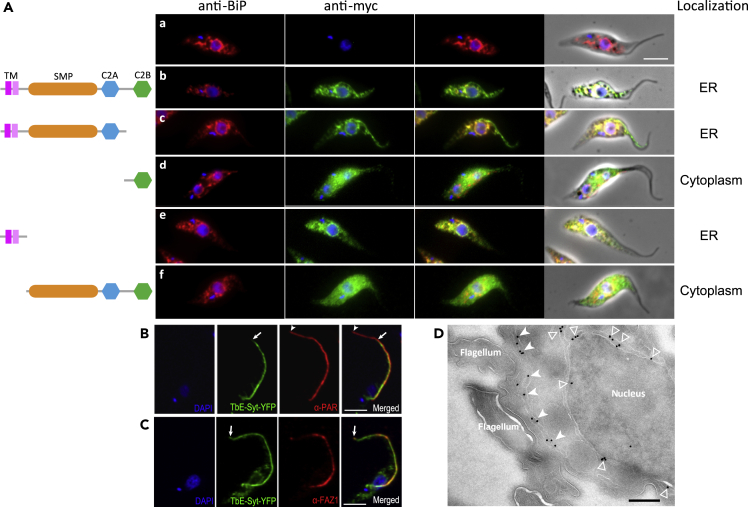
TbE-Syt localizes to the ER (A) TbE-Syt colocalizes with BiP at the ER via its N-terminal transmembrane domain. *T. brucei* procyclic form cells (parental, a) and those expressing various truncations of myc-tagged TbE-Syt (b−f) were probed by immunofluorescence with BiP (red) and anti-myc (green) antibodies. Kinetoplasts and nuclei were stained with DAPI. Scale bar, 5 μm. (B and C) Cells stably expressing TbE-Syt-YFP were stained with DAPI for DNA and labeled with anti-PAR (α-PAR) antibodies for the entire flagellum (B) or with anti-FAZ1 (α-FAZ1) for the FAZ (C). Images were acquired with an LSM510 Meta confocal microscope. The tips of the FAZ and the flagellum are marked by arrows and arrowheads, respectively. Scale bars, 5 μm. (D) Immunogold labeling of TbE-Syt-YFP cells using anti-GFP antibodies. Gold particles were associated both with the central ER around the nuclear envelope (open arrowheads) and with the FAZ-associated ER beneath the flagellum (arrowheads). Scale bar, 200 nm. See also Figures S1–S3.

BiP, which is a molecular chaperone located in the ER lumen, was chosen as an ER marker (Bangs et al., 1993) (Figure 2A, panel a). Co-immunolabeling of BiP and TbE-Syt-myc demonstrated that TbE-Syt co-localizes with BiP at the ER with a Pearson's coefficient (PC) of 0.93 ± 0.04 (Figures 2A, panel b, and S1C). Similarly, TbE-Syt-YFP also localized to the ER in the cell (Figure S2). The C2B domain was not involved in TbE-Syt localization as its deletion or mutation did not change the ER localization of the protein (the PCs were 0.89 ± 0.06 and 0.89 ± 0.10 for T1 and mutC2B, respectively) (Figures 2A, panel c, and S1C, T1 and mutC2B). As expected, when C2B was expressed alone, it was cytosolic (Figures 2A, panel d, and S1C, T2). Notably, although the TM alone co-localized with BiP at the ER (PC 0.96 ± 0.02), it disappeared in the extracted cytoskeletons (Figures 2A, panel e, and S1C, T3). Conversely, the construct lacking the TM was completely cytosolic (Figures 2A, panel f).

Besides the central ER that is connected to the nuclear envelope, the same as in canonical eukaryotes, trypanosomes have an extra unique flagellar attachment zone (FAZ)-associated cortical ER (Sherwin and Gull, 1989; Vickerman, 1969). In detergent-extracted cells (i.e., cytoskeletons), labeling of full-length TbE-Syt-myc remained along the flagellum up to the anterior end of the cell body (Figure S1C) and was detected in the cytoskeleton fraction by WB (Figure S1B). We therefore further checked whether TbE-Syt localizes to the FAZ-associated ER using another *T. brucei* cell line stably expressing C-terminally YFP-tagged TbE-Syt. Two antibodies, anti-PFR2 (L8C4) and anti-FAZ1 (L3B2), were chosen to mark the whole flagellum and the FAZ, respectively (Gadelha et al., 2005; Kohl et al., 1999). Our immunofluorescence data showed that TbE-Syt overlapped with PFR2 along the flagellum, but the signal did not reach the flagellar tip (Figure 2B). TbE-Syt overlapped with FAZ1 along the entire FAZ, however (Figure 2C). This is consistent with the FAZ localization of TbE-Syt reported in a high-throughput screening database where the majority of C-terminally mNeonGreen-tagged TbE-Syt was observed in the FAZ area, with some weak signals forming reticulated patches in the cytoplasm (Dean et al., 2017) (Figure S3).

To further verify its association with the ER, we checked TbE-Syt localization at the ultrastructural level by immuno-gold labeling. The results showed that TbE-Syt localized to both the FAZ-associated ER beneath the flagellum and the central ER around the nuclear envelope (Figure 2D). Overall, our data confirm that TbE-Syt localizes primarily to the FAZ-associated ER in *T. brucei*, and correct localization is mainly mediated by its N-terminal TM domains.

### Homology modeling suggests that TbE-Syt-SMP is capable of binding lipids

**Figure 3:**
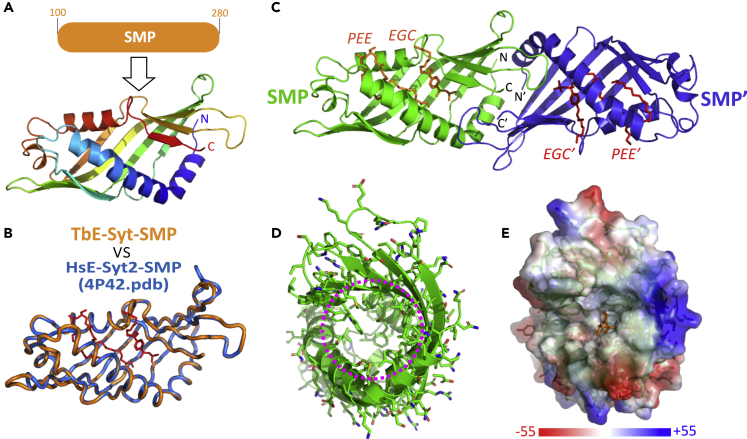
Homology modeling of the SMP domain of TbE-Syt (A) Modeling of the SMP domain (aa100−280) by SWISS-MODEL using the crystal structure of HsE-Syt2 (PDB: 4P42) as the template. (B) TbE-Syt-SMP model (orange) superimposed onto the modeling template (marine). The lipid molecules in the HsE-Syt2 structure are shown as red sticks. (C) Inferred dimeric assembly of TbE-Syt-SMP based on the crystal structure of HsE-Syt2. (D) End view of TbE-Syt-SMP in (A) seen from the right side. The interior of the structure (purple circle) contains mostly hydrophobic residues. (E) Electrostatic surface of TbE-Syt-SMP in the same orientation as in (D). Lipid molecules extracted from the superimposed HsE-Syt2 structure are shown as orange sticks. All images were prepared using PyMOL. See also Figure S4.

The SMP domain is the signature element of all E-Syts. However, none of the TbE-Syt constructs containing the SMP domain we generated produced soluble recombinant protein, which prevented us from carrying out *in vitro* biochemical and structural studies on this critical domain. To understand the structure and function of TbE-Syt-SMP, we turned to bioinformatics analyses to generate a homology model for it. The top hit identified by the protein structure homology-modeling program SWISS-MODEL Workspace (Waterhouse et al., 2018) was the crystal structure of the SMP domain of HsE-Syt2, which was solved to 2.4 Å resolution with bound phospholipids (PDB: 4P42) (Schauder et al., 2014). We chose this crystal structure, which shares 20.45% identity with TbE-Syt-SMP, as the template for our homology modeling (Figures 3A and S4A).

The resulting homodimeric model was of high quality based on the confidence evaluation and local similarity to the template (Figures S4B and S4C). Superimposing the TbE-Syt-SMP model onto the template structure of HsE-Syt2-SMP showed an excellent agreement (Figure 3B). The homodimeric TbE-Syt-SMP model, which was generated using the quaternary structure prediction option of SWISS-MODEL (Bertoni et al., 2017) with lipids extracted from the superimposed template structure, predicted that the lipid molecules would bind in a deep cleft between the long α helix and the edge of the extended β sheet (Figure 3C). This cleft consists almost exclusively of hydrophobic residues that are highly conserved in all trypanosomes, which suggests that TbE-Syt-SMP has the capability of binding lipids in a similar fashion to the SMP domain of HsE-Syt2 (Figures 3D and 3E).

### The TbE-Syt C2B domain contains a conserved Ca^2+^-binding site

**Figure 4:**
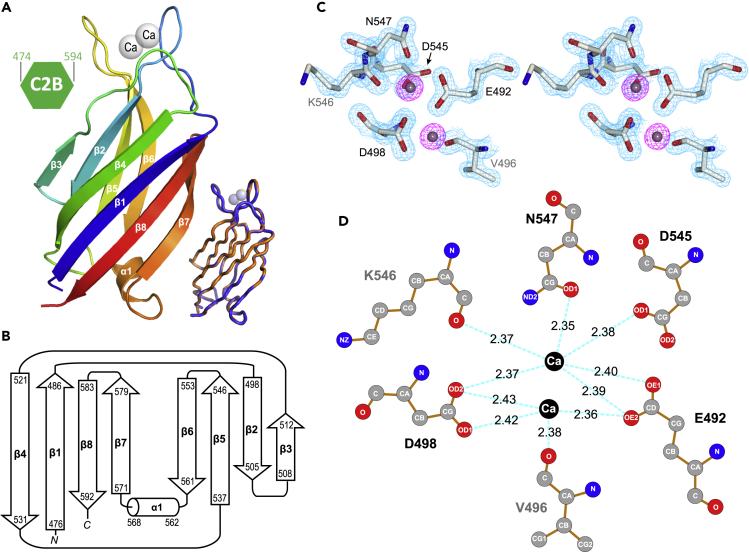
Crystal structure of the C2B domain of TbE-Syt (A) Ribbon diagram of the TbE-Syt-C2B crystal structure color-ramped from blue at the N terminus to red at the C terminus. Secondary structures and the two bound Ca^2+^ ions (gray spheres) are labeled. The two molecules in the asymmetric unit are shown superimposed in the lower right corner. (B) Secondary structure diagram of TbE-Syt-C2B, with the residue ranges of each structural element indicated. (C) Stereo view of the 2*Fo*-*Fc* map around the Ca^2+^-binding site contoured at 2 *σ* level. Electron densities around the Ca^2+^ ions are highlighted in purple. The plot was produced using CCP4mg (McNicholas et al., 2011). (D) Hydrogen bonds between the Ca^2+^ ions and coordinating residues in TbE-Syt-C2B. The plot was generated using LigPlot+ (Laskowski and Swindells, 2011). See also Figure S5.

In contrast to mammalian E-Syts that have at least three C2 domains, TbE-Syt contains only two C2 domains. To further characterize the structure and function of TbE-Syt, we first determined a crystal structure for its C2B domain (Figure 4A). The structure was solved to 1.5 Å resolution using the selenium single-wavelength anomalous diffraction *de novo* phasing method (Table 1). There are two molecules *per* asymmetric unit, both of which contain aa474-594 of TbE-Syt. The two molecules are essentially identical, with an average root-mean-square deviation (RMSD) of 0.25 Å for all aligned backbone atoms.

**Table 1:** Data collection and refinement statistics

**Data collection**	

Space group	P2_1_2_1_2_1_
Wavelength (Å)	0.873
Cell dimensions	
*a*, *b*, *c* (Å)	54.91, 57.53, 84.57
Resolution (Å)	50.0–1.50 (1.55–1.50)^a^
Total reflections	232,968 (23,835)
Unique reflections	43,464 (4,297)
Multiplicity	5.4 (5.5)
R-_merge_	0.083 (0.978)
R_-meas_	0.092 (1.035)
R_-pim_	0.039 (0.429)
CC(1/2)	0.998 (0.644)
CC^a^	0.999 (0.885)
Mean I/*σ(I)*	10.39 (1.37)
Completeness (%)	99.6 (99.8)
Wilson B-factor	17.08

**Refinement**	

Resolution (Å)	20–1.50
Number of reflections	43,445
*R*_work_/*R*_free_ (%)	16.97/19.13
No. atoms	
Protein	1,986
Water	353
Ligand/ion	16
*B*-factors	
Protein	22.7
Water	36.7
Ligand/ion	24.7
RMSD	
Bond lengths (Å)	0.012
Bond angles (°)	1.221
Ramachandran plot	
Favored (%)	100.00
Allowed (%)	0
Outlier (%)	0

a Values in parentheses are for the highest resolution shell.

The structure showed that TbE-Syt-C2B adopts a type II topology, consisting of eight β strands that form two sheets and a short α helix in the loop connecting β6 and β7 (Figure 4B). Additionally, there are two tightly bound Ca^2+^ ions, which are coordinated by a few charged residues in two extended loops on the opposite end from the helix (Figure 4C). The interaction is mediated by a network of hydrogen bonds between the Ca^2+^ ions and the side-chain oxygens of residues E492, D498, D545, and N547, which are all highly conserved in trypanosomes (Figure 4D). The main-chain carboxyl oxygens of residues V496 and K546 also form hydrogen bonds with either of the two Ca^2+^ ions. Additionally, there are four stably bound water molecules hydrogen bonded to these two Ca^2+^ ions (Figure S5).

### TbE-Syt-C2B binds lipids in both Ca^2+^- and PI(4,5)P_2_-dependent manners

**Figure 5:**
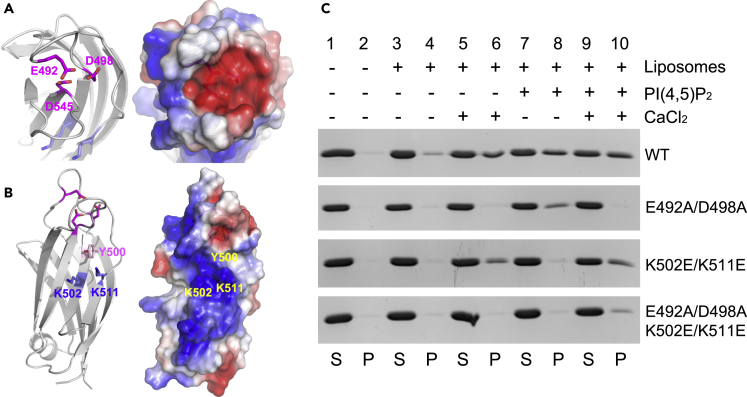
TbE-Syt-C2B binds lipids using two distinct sites (A) Ribbon (left) and electrostatic (right) plots of the Ca^2+^-binding acidic patch in the TbE-Syt C2B domain. The three highly conserved acidic residues in the core of the patch are shown as sticks and labeled. Ca^2+^ ions are omitted for clarity. (B) Side view of TbE-Syt-C2B with the two basic residues in the center of the positively charged patch labeled. (C) Liposome pelleting assays of wild-type (WT) and three mutants of TbE-Syt-C2B in the presence or absence of PI(4,5)P_2_ and/or CaCl_2_. S: supernatant; P: pellet.

Similar to the Ca^2+^-binding site in the C2A domain of HsE-Syt2, TbE-Syt-C2B also has an extremely acidic “crater” on its surface (Figure 5A). Previous studies showed that the C-terminal C2 domain of mammalian E-Syt2 and E-Syt3 interacts with the plasma membrane via a basic surface patch (Giordano et al., 2013). In the TbE-Syt-C2B structure there is also a basic patch on β2 and β3, which contains residues K502 and K511 at its center (Figure 5B). Additionally, there is a highly conserved aromatic residue, Y500, at the deepest point of this curved patch. Both the basic patch and the conserved Y500 residues are reminiscent of the C2 domain of PKCα that associates with PI(4,5)P_2_ via a similar basic patch (Guerrero-Valero et al., 2009).

To check whether TbE-Syt-C2B interacts with lipids through either or both the Ca^2+^-binding site and the basic patch, we generated two types of mutations. In one mutant, we abolished Ca^2+^ binding by mutating residues E492 and D498, which are simultaneously hydrogen bonded with both Ca^2+^ ions, to alanine (E492A/D498A). In another mutant, we changed the two lysine residues at the center of the basic patch to opposite charges, i.e., K502E/K511E. Additionally, we generated a combined mutant for all these residues, E492A/D498A/K502E/K511E.

We used *in vitro* liposome binding assays to investigate whether TbE-Syt-C2B binds liposomes and, if so, whether any of the mutants affect this interaction. Purified wild-type and mutant proteins were incubated with sucrose-loaded vesicles containing different molar ratios of lipids and pelleted at high speed to separate the liposome-bound and unbound fractions of the proteins. SDS-PAGE analyses of the supernatant (unbound) and pellet (liposome-bound) fractions showed that wild-type TbE-Syt-C2B bound liposomes very weakly in the absence of both Ca^2+^ and PI(4,5)P_2_ (Figure 5C, WT: lane 4). However, addition of either Ca^2+^ or PI(4,5)P_2_ to the reaction showed robust binding (Figure 5C, WT: lanes 6 and 8). Notably, protein bound to liposomes was slightly more abundant when both Ca^2+^ and PI(4,5)P_2_ were present (Figure 5C, WT: lane 10). The mutant E492A/D498A abolished only the Ca^2+^-dependent lipid binding, but not PI(4,5)P_2_-mediated binding (Figure 5C, lane 6 versus lane 8). Interestingly, addition of Ca^2+^ abolished the PI(4,5)P_2_-mediated liposome binding of this mutant even though PI(4,5)P_2_ was present on the liposomes (lane 10). This was probably because excessive Ca^2+^ ions would bind to the negatively charged head groups of PI(4,5)P_2_ and thus shielded the interaction of the basic patch with the lipid. The converse effect was observed for the basic patch mutant (K502E/K511E), which retained the Ca^2+^-dependent liposome binding while completely losing PI(4,5)P_2_-lipid binding ability (Figure 5C, lane 6 versus lane 8). This indicated that TbE-Syt-C2B binds liposomes in two different fashions. Indeed, only upon mutating both Ca^2+^-binding and the central basic patch residues (E492A/D498A/K502E/K511E) was the liposome binding of TbE-Syt-C2B completely abolished.

### TbE-Syt-C2A lacks the Ca^2+^-binding site but retains the PI(4,5)P_2_-binding patch

**Figure 6:**
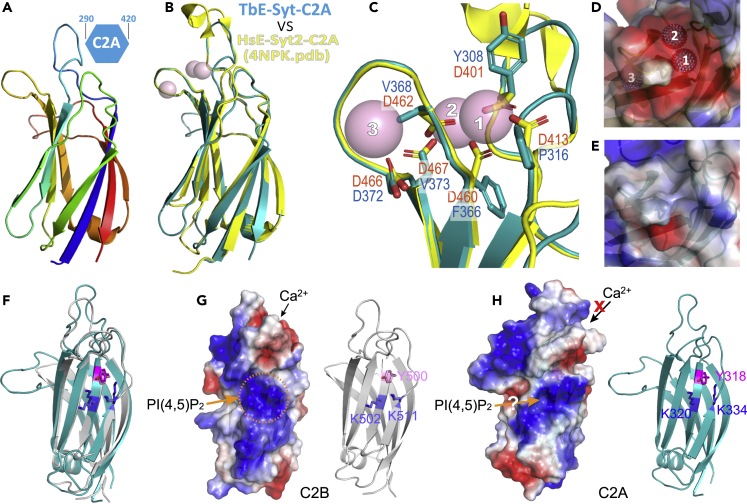
Homology modeling of the TbE-Syt C2A domain (A) Modeling of TbE-Syt-C2A (aa290−420) by SWISS-MODEL using the homologous structure of the HsE-Syt2-C2A (PDB: 4NPK) as the template. (B) TbE-Syt-C2A model (cyan) superimposed onto the modeling template (yellow). The three Ca^2+^ ions in the template structure are shown as pink spheres. (C) Close-up view of the Ca^2+^-binding site of the superimposed structures in (B). Residues in HsE-Syt2 coordinating Ca^2+^ ions (pink spheres) are shown as orange sticks, and corresponding residues in TbE-Syt are shown as teal sticks. (D) Electrostatic plot of the Ca^2+^-binding site of HsE-Syt2, with the three Ca^2+^ ions shown as labeled spheres. (E) Corresponding region in TbE-Syt-C2A in the same orientation as in (D) and with the same electrostatic scale. The putative Ca^2+^-binding site is mostly hydrophobic in TbE-Syt-C2A. (F) Superimposed ribbon diagrams of the C2A model and the C2B crystal structure of TbE-Syt. The three critical residues in the patches for PI(4,5)P_2_-mediated lipid binding are shown as sticks. (G) Electrostatic plot and ribbon diagram of TbE-Syt-C2B. Ca^2+^ ions bind to the acidic patch at the top end, and the PI(4,5)P_2_-binding site is encircled in orange. (H) Dual plots of TbE-Syt-C2A in the same orientation as (F). The putative Ca^2+^-binding site is hydrophobic and not able to bind Ca^2+^, the PI(4,5)P_2_-binding site is highly conserved. This suggests that TbE-Syt-C2A could potentially binds lipids in the PI(4,5)P_2_-mediated manner. See also Figure S6.

Previous studies have shown that the C2A domain in all mammalian E-Syts binds Ca^2+^ and is involved in lipid interaction (Min et al., 2007; Xu et al., 2014). We wondered whether TbE-Syt-C2A functions similarly. We tried to express recombinant TbE-Syt-C2A, either alone or together with C2B, SMP, or both, but did not succeed in obtaining soluble proteins. We thus tried to understand its structure and function by homology modeling. One of the top hits recognized by SWISS-MODEL (Waterhouse et al., 2018) was the crystal structure of HsE-Syt2-C2A (PDB: 4NPK) (Xu et al., 2014), which shares ∼17% identity with TbE-Syt-C2A. High local similarity of the final model and consistent alignment of secondary structures between the model and the template suggested that it is a convincing homology model (Figures 6A and S6). Superimposing the TbE-Syt-C2A model onto the HsE-Syt2-C2A crystal structure also showed an overall very good agreement (Figure 6B).

The previously reported crystal structure of HsE-Syt2-C2A contains three tightly bound Ca^2+^ ions coordinated by six Asp residues from two connecting loops via an extensive hydrogen bond network. This site is responsible for the binding of HsE-Syt2-C2A to lipids (Xu et al., 2014). Surprisingly, close examination of the putative Ca^2+^-binding site of TbE-Syt-C2A in the superimposed structures revealed that five of the six Asp residues in HsE-Syt2-C2A are substituted by hydrophobic residues in TbE-Syt-C2A (Figure 6C). Consequently, the concave acidic patch for Ca^2+^ binding present in HsE-Syt2-C2A (Figure 6D) was replaced by a saddle-like hydrophobic area in TbE-Syt-C2A (Figure 6E). This suggests that the C2A domain in TbE-Syt is unlikely to bind Ca^2+^.

Previous studies showed that C2A and C2B of HsE-Syt2 share a similar conformation (Xu et al., 2014). We superimposed the homology model of TbE-Syt-C2A onto the crystal structure of TbE-Syt-C2B and found that not only was their overall conformation very similar but also the PI(4,5)P_2_-binding site was present in both C2 domains (Figures 6F and 6G). As shown above, we have found that TbE-Syt-C2B interacts with lipids in both Ca^2+^- and PI(4,5)P_2_-mediated fashions (Figure 5C). The highly conserved basic patch on TbE-Syt-C2A suggests that, despite lacking a Ca^2+^-binding site, it might be still involved in lipid binding via the PI(4,5)P_2_-dependent mode (Figure 6H).

### TbE-Syt-C2A and C2B are connected by a long flexible loop

Previous structural studies revealed that the first two C2 domains, i.e., C2A and C2B, of HsE-Syt2 shares high structural similarities with each other (Xu et al., 2014). A high-resolution NMR structure of the last C2 domain of HsE-Syt2, i.e., C2C, has also been determined (PDB: 2DMG). We therefore compared the structure of TbE-Syt-C2B with that of the three C2 domains of HsE-Syt2. TbE-Syt-C2B adopts the same topology as HsE-Syt2-C2A and C2B, i.e.*,* type II, whereas HsE-Syt2-C2C has a distinct type I topology (Figure S7A). Superimposing TbE-Syt-C2B onto each of the three C2 domains of HsE-Syt2 showed that it is very similar to HsE-Syt2-C2A and C2B, with average RMSDs of 1.05 Å and 0.75 Å, respectively, but substantially deviated from C2C, with an RMSD of 3.57 Å (Figures S7B and S7C). This further confirms that the C2A and C2B domains in TbE-Syt are equivalent to C2A and C2B of HsE-Syt2.

**Figure 7:**
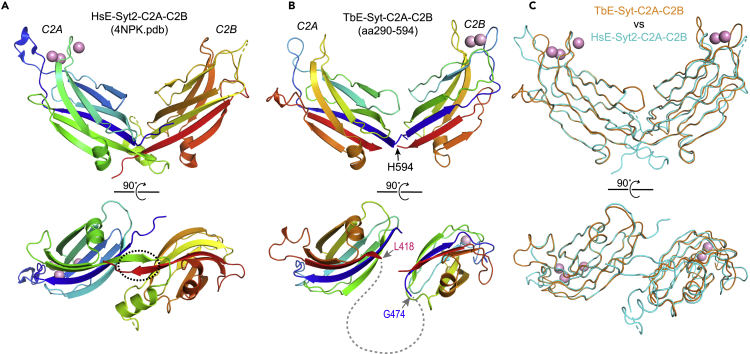
The C2A and C2B domains of TbE-Syt are connected by a long flexible loop (A) Ribbon diagram of HsE-Syt2-C2A-C2B (PDB: 4NPK), shown in two orientations. The anti-parallel interaction of the last β strand from both domains is indicated with a dashed oval in the lower orientation. (B) Ribbon diagrams of TbE-Syt-C2A-C2B in the same orientations as in (A). TbE-Syt-C2A is the homology model, and C2B is the crystal structure. H594 is the last residue at the C terminus of TbE-Syt. The last β strands in both TbE-Syt-C2A and C2B are much shorter than those in HsE-Syt2-C2A and C2B and thus not able to make an inter-strand interaction as in HsE-Syt2-C2A-C2B. The long flexible linker is shown as a dashed line. (C) TbE-Syt-C2A-C2B and HsE-Syt2-C2A-C2B as shown in (A) and (B), superimposed. See also Figures S7 and S8.

HsE-Syt2-C2A and C2B fold into a compact V-shaped conformation, which is stabilized by the antiparallel interaction of the C termini of the last β strands on these domains (Xu et al., 2014) (Figure 7A). Structural comparison with HsE-Syt2-C2A-C2B showed that the last β strands of both TbE-Syt-C2A and C2B are too short to allow similar inter-strand interaction between them (Figures 7B and 7C). Therefore, the linker between TbE-Syt-C2A and C2B, which consists of 55 residues (aa419−473), likely forms a long flexible loop. The predicted flexibility of the loop is supported by the folding analysis on TbE-Syt (Figure S8). Local dynamic state of each residue in TbE-Syt was quantitatively evaluated using ODiNPred, which uses a deep neural network trained on experimental NMR-derived *Z* scores and has been shown superior in predicting disordered loops (Dass et al., 2020). ODiNPred strongly suggests that the region spanning aa418−474 in TbE-Syt is exclusively disordered (Figure S8A).

## Discussion

We report here our structural characterizations of an uncharacterized E-Syt from *T. brucei*. Our bioinformatics analyses showed that, similar to all known mammalian E-Syts, TbE-Syt also has two TM motifs at its N terminus and a central SMP domain (Figure 1). However, it contains only two C2 domains, in contrast to at least three C2 domains in all other reported E-Syts in animals, plants, and yeast (Craxton, 2007; Giordano et al., 2013; Min et al., 2007; Toulmay and Prinz, 2012). This makes TbE-Syt the shortest E-Syt identified to date.

Despite testing several RNAi constructs, RNAi against TbE-Syt in both procyclic and bloodstream forms of *T. brucei* did not decrease the expression of the protein (data not shown), which prevented us from further analyzing its cellular function. Previous systematic high-throughput RNAi screening data showed that depletion of TbE-Syt had no significant effect in bloodstream form *T. brucei*, but deleteriously affected procyclic forms (Figure S9) (Alsford et al., 2011). This suggests that TbE-Syt might play a vital role under certain conditions. Indeed, whereas no severe defects were observed upon their deletion in many organisms (Manford et al., 2012; Saheki et al., 2016; Sclip et al., 2016; Toulmay and Prinz, 2012; Tremblay and Moss, 2016), some specific defects have been observed in yeast, plants, and animals (Aguilar et al., 2007; Levy et al., 2015; Saheki et al., 2016). It is noteworthy that orthologs of TbE-Syt show high synteny in all pathogenic trypanosomes, further suggesting that it is an important gene that has been conserved over evolutionary time.

It was shown previously that in other organisms, including yeast and mammals, E-Syts are selectively concentrated in the cortical ER adjacent to the plasma membrane. This localization can be either constitutive or under the control of the cytosolic Ca^2+^ level (Chang et al., 2013; Giordano et al., 2013; Idevall-Hagren et al., 2015; Manford et al., 2012; Toulmay and Prinz, 2012). Our *in vivo* localization data showed that TbE-Syt localizes to both the central ER close to the nuclear envelope and the FAZ-associated cortical ER beneath the flagellum (Figure 2D). Localization to the central ER was mediated exclusively by the TM motifs as the TM region itself was sufficient to target to the central ER (Figures 2A and S1). However, the TM motifs alone did not localize to the FAZ area and, unlike other longer constructs including full-length and TM-SMP-containing TbE-Syt, were removed in the extracted cells (Figure S1C, T3 construct). It is noteworthy that most tagged TbE-Syts used in our studies were overexpression constructs, which often yield a much higher expression level than that of the endogenous proteins. Notably, the expression levels were also different among constructs, preventing quantitative comparison in WB and immunofluorescence experiments. Tube-like connections have been observed between the FAZ-associated ER and the central ER around the outer nuclear membrane (Lacomble et al., 2009). At an elevated expression level, a fraction of TbE-Syt might also redistribute to the central ER through such tube-like connections. Notably, TbE-Syt has been independently found to localize to the FAZ area and associate with CIF1, a protein present at the distal tip of the newly assembled FAZ (Zhou et al., 2018). Therefore, under physiological conditions TbE-Syt likely localizes only to the FAZ-associated cortical ER apposed to the plasma membrane.

It was reported previously that inter-membrane lipid transfer by HsE-Syt2 is through its SMP domain as the carrier of phospholipids (Schauder et al., 2014). Homology modeling analyses showed that TbE-Syt-SMP has a similar deep hydrophobic cleft that could accommodate lipid molecules as in HsE-Syt2 (Figure 3). Similar to HsE-Syt2, the SMP of TbE-Syt could also form an end-to-end homodimer based on our bioinformatics analyses. Both the putative lipid-binding cleft in TbE-Syt-SMP and the hydrophobic dimeric interface are highly conserved in all trypanosome homologs, suggesting that TbE-Syt might employ a similar mechanism to carry and transfer lipids as that reported for HsE-Syt2.

We have determined a high-resolution crystal structure for the C-terminal C2 domain of TbE-Syt (Figure 4), which was designated as C2B based on its high structural similarity to HsE-Syt2-C2B (Figure S7). Our structure-based mutagenesis studies revealed that TbE-Syt-C2B bound lipids in both Ca^2+^- and PI(4,5)P_2_-dependent manners via an acidic patch and an extended basic patch, respectively (Figure 5). Although disruption of one site did not abolish lipid binding of the other, wild-type protein bound liposomes much more abundantly than either mutant that abolished one of the two binding sites. Therefore, the two sites seem to work cooperatively in lipid binding, similar to what was reported for C2 domains of Syt1 (Li et al., 2006; Radhakrishnan et al., 2009; van den Bogaart et al., 2012). For TbE-Syt-C2A, homology modeling suggested that it lacks nearly all acidic residues at the putative Ca^2+^ binding site. However, TbE-Syt-C2A retains a conserved basic patch similar to that on TbE-Syt-C2B (Figure 6). This suggests that TbE-Syt-C2A may use only the basic patch to interact with the negatively charged head groups of PI(4,5)P_2_ enriched on the plasma membrane.

With only two C2 domains, TbE-Syt is the shortest E-Syt identified to date. This may correlate with the unique MCS between the FAZ-associated ER and the plasma membrane in *T. brucei*. The FAZ in trypanosomes forms an adhesion structure between the flagellar and the plasma membrane, with one portion of the FAZ filament tightly associated with the cortical ER (Lacomble et al., 2009; Vickerman, 1969). The specialized FAZ in trypanosomes makes the FAZ-associated ER in a close and constitutive contact with the plasma membrane. In such a case, a short tether like TbE-Syt would be sufficient to bridge the two apposed membranes to allow efficient lipid transfer between the FAZ-associated cortical ER and the plasma membrane. Furthermore, in contrast to other organisms, including yeast, plants, and animals, which all have several types of E-Syts, trypanosomes have only a single copy of E-Syt. This might also correlate with the specialized function of TbE-Syt at the MCS between the FAZ-associated ER and the plasma membrane in trypanosomes.

**Figure 8:**
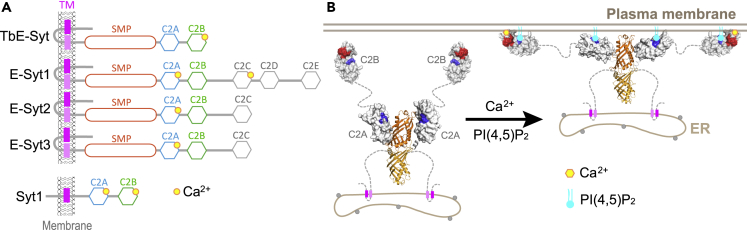
Structural comparison and hypothetical working model of TbE-Syt in lipid transferring (A) Comparison of the domain arrangement of TbE-Syt and mammalian E-Syts and Syt1. All C2 domains in Syt1 bind Ca^2+^, whereas only some of the C2 domains in the E-Syts bind Ca^2+^. TbE-Syt is distinct from all known mammalian E-Syt1, 2, and 3 in that it contains only two C2 domains. Furthermore, TbE-Syt-C2B binds Ca^2+^ but TbE-Syt-C2A does not, which is the opposite of E-Syt1-3. (B) Hypothetical model illustrating how the ER-anchored TbE-Syt might attach to the plasma membrane via both Ca^2+^- and PI(4,5)P_2_-mediated interactions. This would tether the cortical ER to the plasma membrane to allow the lipid-carrying SMP dimer to shuttle lipids between the two apposed membranes. See also Figure S9.

Based on the unique domain organization and distinct structure of TbE-Syt compared with mammalian E-Syts and Syt1 (Figure 8A), we propose a working model for how TbE-Syt might mediate lipid transfer between the cortical ER and the plasma membrane in trypanosomes (Figure 8B). Two TbE-Syt molecules form a homodimer through the end-to-end interaction of their SMP domains. The C2A domain is connected to the SMP via a short linker (aa280−290), which is predicted to be ordered by ODiNPred (Figure S8A). Consistently, homology modeling based on the crystal structure of HsE-Syt-SMP-C2A (PDB: 4NPK) generated a compact structure of TbE-Syt-SMP-C2A (Figure S8B). To generate MCSs between the ER and the plasma membrane, the dimeric TbE-Syt is first anchored on the ER membrane via the N-terminal TM domains. The two C2B domains at the distal end of the structure would make initial contact with the plasma membrane both via the acidic patch in a Ca^2+^-dependent manner and via the basic patch to interact with PI(4,5)P_2_. It is noteworthy that the basic residues (K502, K511) in the PI(4,5)P_2_-binding site on TbE-Syt-C2B are not as conserved as those acidic residues in the Ca^2+^-binding site in other trypanosome species (Figure 1B), suggesting that the Ca^2+^-binding site is the major and universal element for TbE-Syt-C2B to interact with the plasma membrane.

In contrast, the basic patch on TbE-Syt-C2A is highly conserved throughout all trypanosomes. Given that it lacks the Ca^2+^-binding site, TbE-Syt-C2A would dock onto the plasma membrane solely in a PI(4,5)P_2_-dependent fashion. Interestingly, the putative PI(4,5)P_2_-binding site in the homologous model of TbE-Syt-C2A is orientated inward facing the tubular SMP (Figure 8B, left). In such case the hinge between the SMP and C2A domains would have to be partially opened to allow the exposure of the basic patch on the C2A domain to interact with the acidic head groups of PI(4,5)P_2_ molecules on the plasma membrane. Notably, the linker connecting C2A and C2B contains a couple of conserved positively charged residues (Figure 1B), whereas high-throughput proteomics studies revealed six phosphorylation sites in this loop, i.e., S437, S441, S443, S447, S459, T460 (Alsford et al., 2011). These suggest that the linker between TbE-Syt-C2A and C2B may play a regulatory role in fine-tuning the interaction between TbE-Syt and the plasma membrane. Altogether, TbE-Syt could thereby tether and staple the FAZ-associated cortical ER onto the plasma membrane to allow the dimeric SMP domains, which could carry multiple lipid molecules in their hydrophobic clefts, to shuttle lipids originating from the ER membrane through their hydrophobic tunnels to the plasma membrane.

### Limitations of the study

Although we have characterized the structure and membrane binding of the C2B domain of TbE-Syt, we could not express and purify its two other domains, i.e., SMP and C2A. This prevents us from revealing their atomic structures and carrying out lipid transfer assays as those previously reported for mammalian E-Syt2.

### Resource availability

#### Lead contact

Further information and requests for resources and reagents should be directed to and will be fulfilled by the lead contact, Gang Dong (gang.dong@meduniwien.ac.at).

#### Materials availability

All unique/stable reagents generated in this study are available from the lead contact without restriction.

#### Data and code availability

The accession number for the crystal structure of TbE-Syt-C2B reported in this paper is PDB: 7A1R.

## Methods

All methods can be found in the accompanying transparent methods supplemental file.

## Supplemental information

Document S1. Transparent methods and Figures S1–S9
