# Supplementary material for: Structural studies of the shortest extended synaptotagmin with only two C2 domains from Trypanosoma brucei
Source: iScience. 2021 Apr 20;24(5):102422. doi: 10.1016/j.isci.2021.102422 (PMC8093936; doi:10.1016/j.isci.2021.102422)
Supplement: Document S1. Transparent methods and Figures S1–S9 [file mmc1.pdf]

iScience, Volume *24*

Supplemental information

Structural studies of the shortest extended synaptotagmin with only two C2 domains from *Trypanosoma brucei*

Emma Stepinac, Nicolas Landrein, Daria Skwarzyńska, Patrycja Wójcik, Johannes Lesigang, Iva Lučić, Cynthia Y. He, Mélanie Bonhivers, Derrick R. Robinson, and Gang Dong

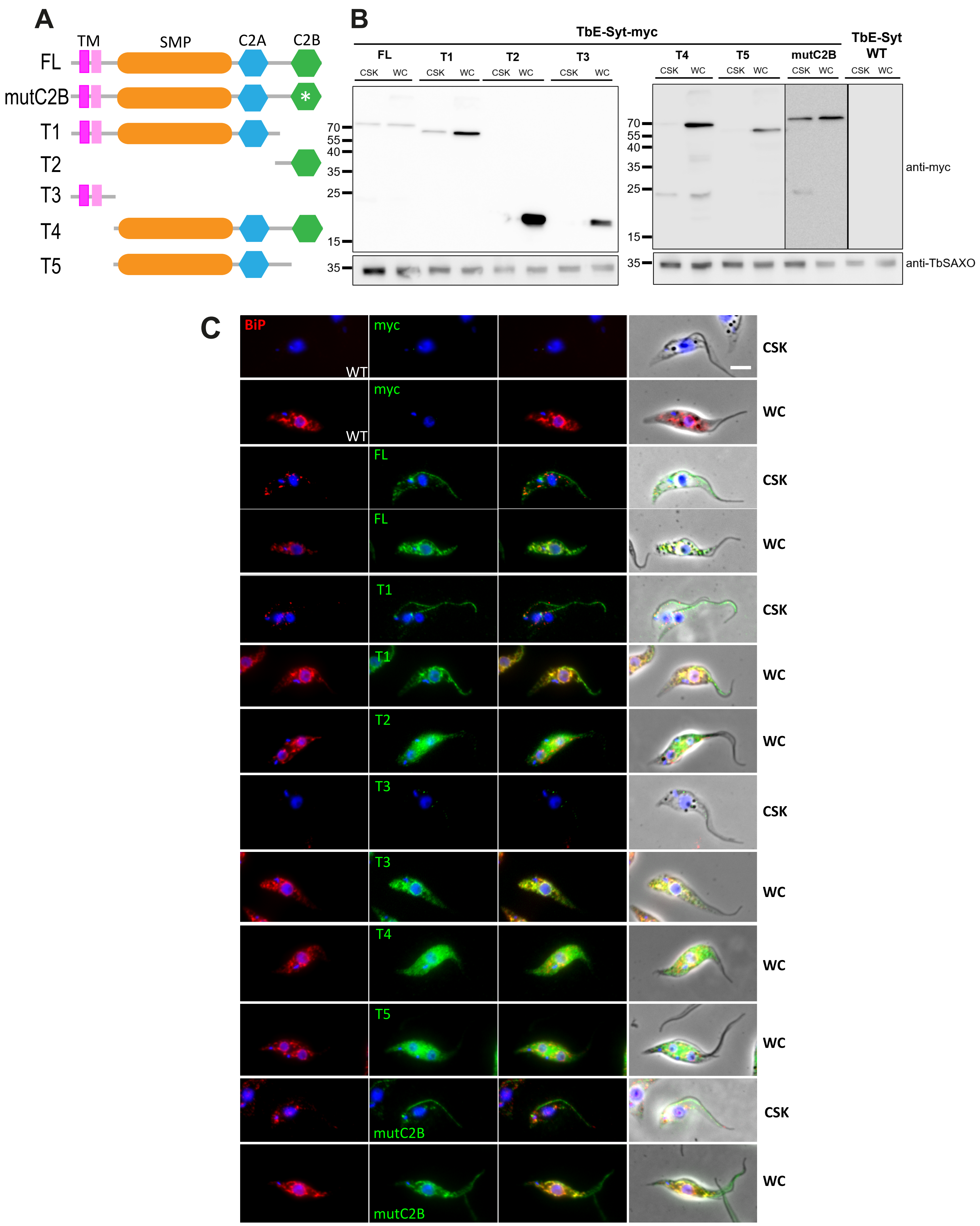

6-24h induction with 10ng/ml tetracycline (T3 with 100ng/ml)
CSK: cytoskeleton extracted cells; **WC:** whole cells

Figure S1. Localization of TbE-Syt at the ER. Related to Figure 2. (**A**) Schematic representations of the C-terminally myc-tagged TbE-Syt constructs, including full-length (FL), mutC2B (E492A/D498A), and five truncations (T1-T5), expressed in procyclic form *T. brucei*. (**B**) Western blot analysis of the level of expression of TbE-Syt-myc constructs in whole cells (WC) and their association with the cytoskeleton in detergent-extracted cells (CSK). Anti-TbSAXO antibodies were used as a loading control. (**C**) Labeling of BiP and myc-tagged TbE-Syt, mutC2B, and truncations T1-T5 in whole cells (WC) and cytoskeletons (CSK). The kinetoplasts and nuclei were stained with DAPI. Scale bar, 5 μm.

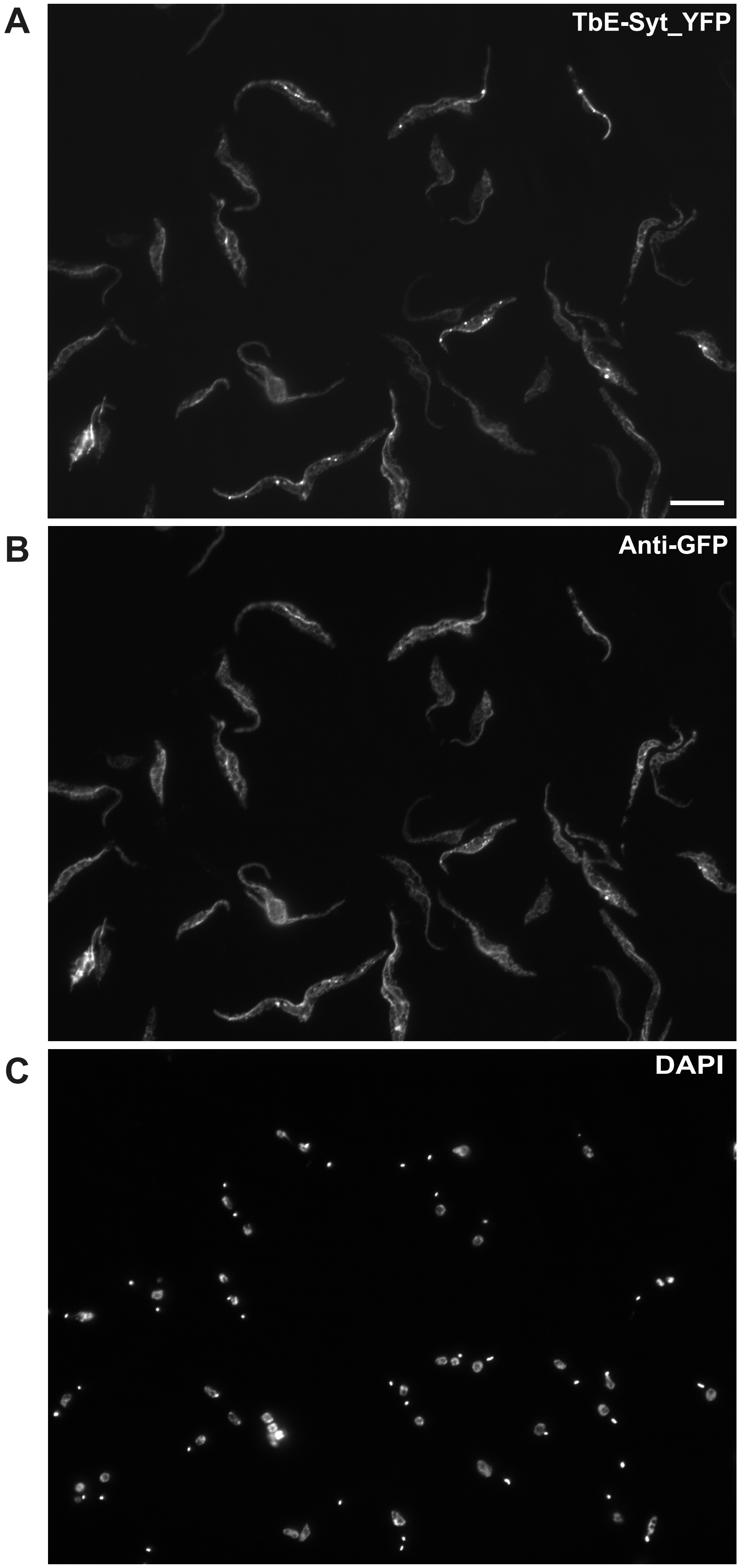

Figure S2. TbE-Syt-YFP localized to both the FAZ-associated ER and the central ER. Related to Figure 2. Note: YFP fluorescence (A) gave slightly weaker but sharper signals than anti-GFP immunofluorescence (B). Scale bar, 10 μm.

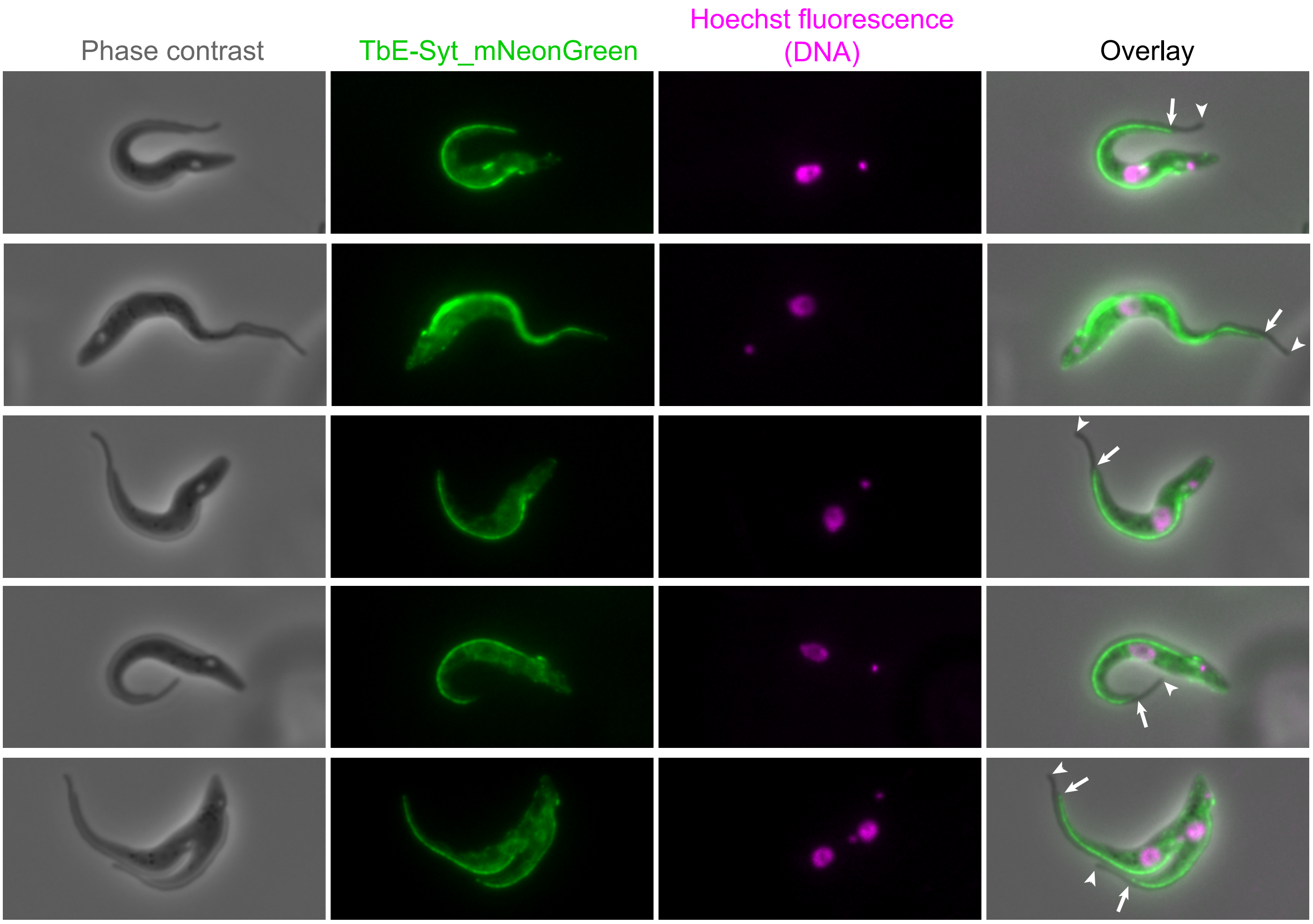

http://tryptag.org/?query=Tb927.10.13740

Figure S3. Location of TbE-Syt in procyclic form *T. brucei* reported in the TrypTag database
Related to Figure 2. Five images with cells expressing TbE-Syt-mNeonGreen were imaged with phase contrast, mNeonGreen fluorescence, and Hoechst 33342 fluorescence. Hoechst 33342 is a fluorescent marker for DNA in the nucleus and kinetoplast (Hoechst). In the overlaid images on the right, the flagellar tips and the tips of the FAZ are marked by arrowheads and arrows, respectively. Images taken with permission from the TrypTag database (http://tryptag.org).

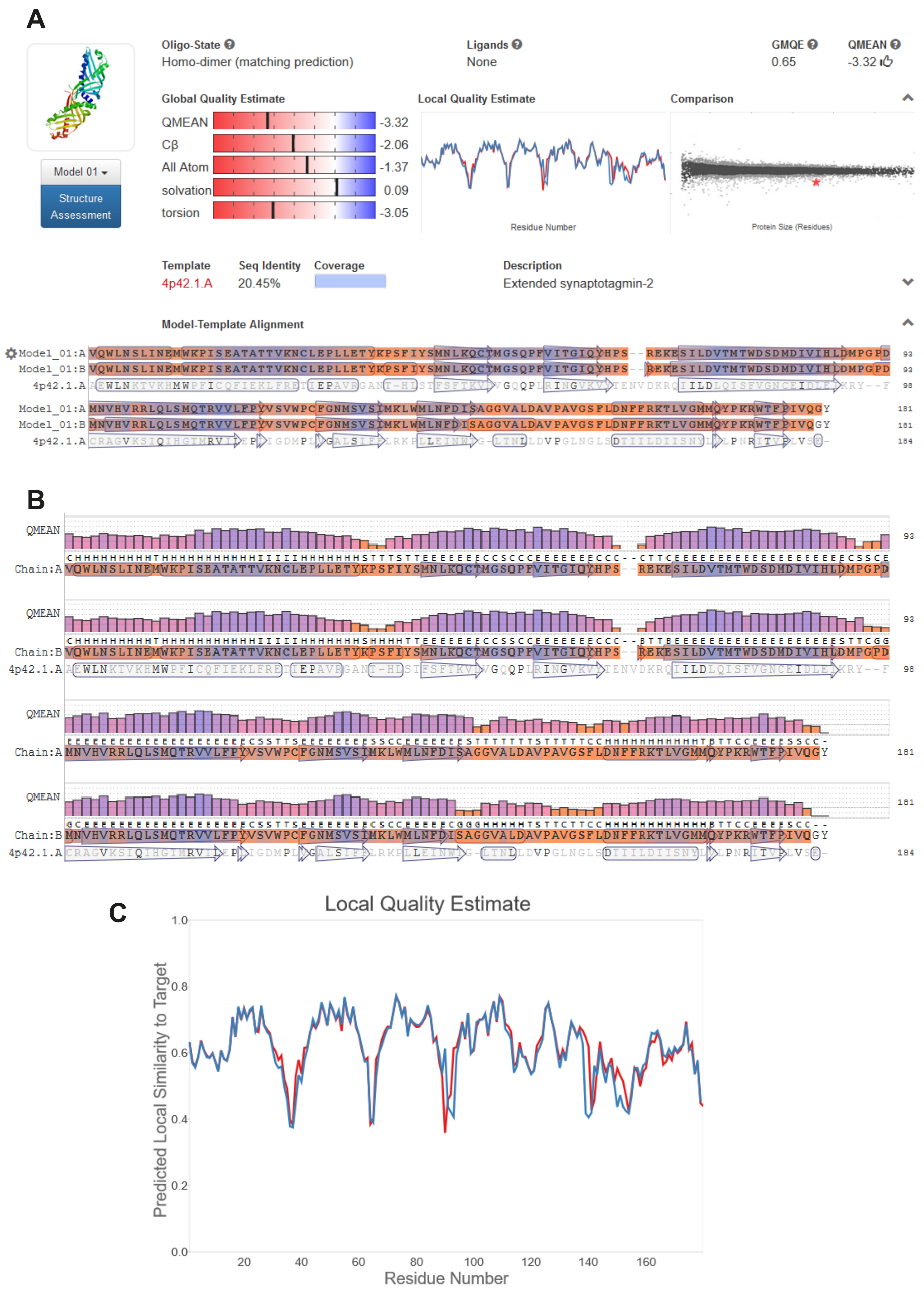

A
Oligo-State
Homo-dimer (matching prediction)
Ligands
None
GMQE
0.65
QMEAN
-3.32
Model 01
Structure Assessment
Global Quality Estimate
QMEAN -3.32
Cβ -2.06
All Atom -1.37
solvation 0.09
torsion -3.05
Local Quality Estimate
Residue Number
Comparison
Protein Size (Residues)
Template
4p42.1.A
Seq Identity
20.45%
Coverage
Description
Extended synaptotagmin-2
Model-Template Alignment
Model_01:A
Model_01:B
4p42.1.A
B
QMEAN
Chain:A
Chain:B
4p42.1.A
C
Local Quality Estimate
Predicted Local Similarity to Target
Residue Number

Figure S4. Homology modeling of the TbE-Syt-SMP dimer. Related to Figure 3. (**A**) Homology modeling of TbE-Syt-SMP by SWISS-MODEL using the HsE-Syt2-SMP structure (4P42.pdb) as the template. (**B**) Alignment of chains A and B of the homodimeric TbE-Syt-SMP model along the template with secondary structures shown in the DSSP mode and individual residues colored based on QMEAN values. (**C**) Local quality estimate for each residue in the final model. The two monomers are colored in red and blue.

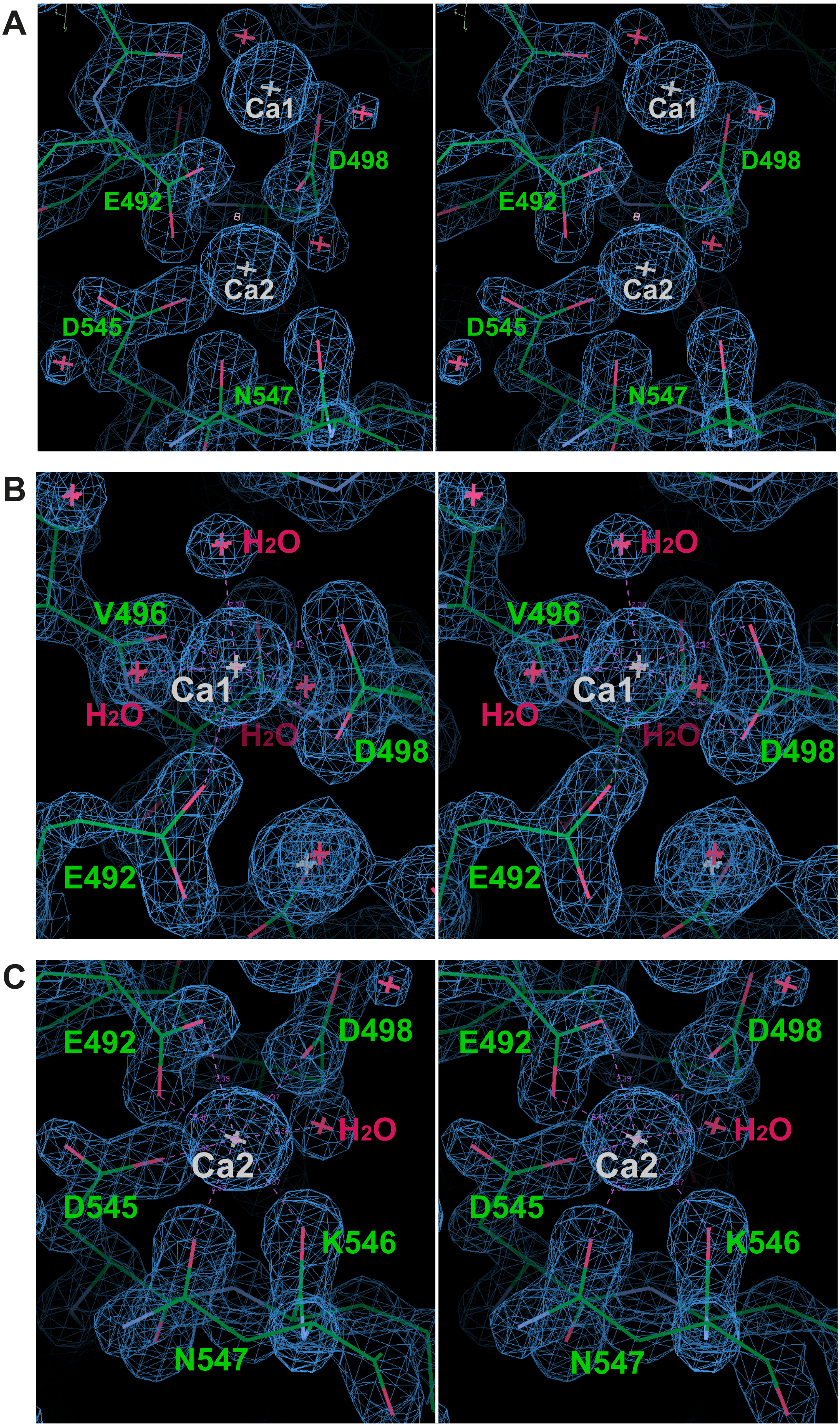

Figure S5. Electron density maps of TbE-Syt-C2B around the two Ca^{2+}-binding sites. Related to Figure 4. Stereo views of the $2F_o$-F_c maps around both (**A**) or each (**B & C**) of the two Ca^{2+}-binding sites. The maps were contoured at 2 σ level. Amino acids and water molecules hydrogen-bonded to the two Ca^{2+} ions are labeled in green and red, respectively.

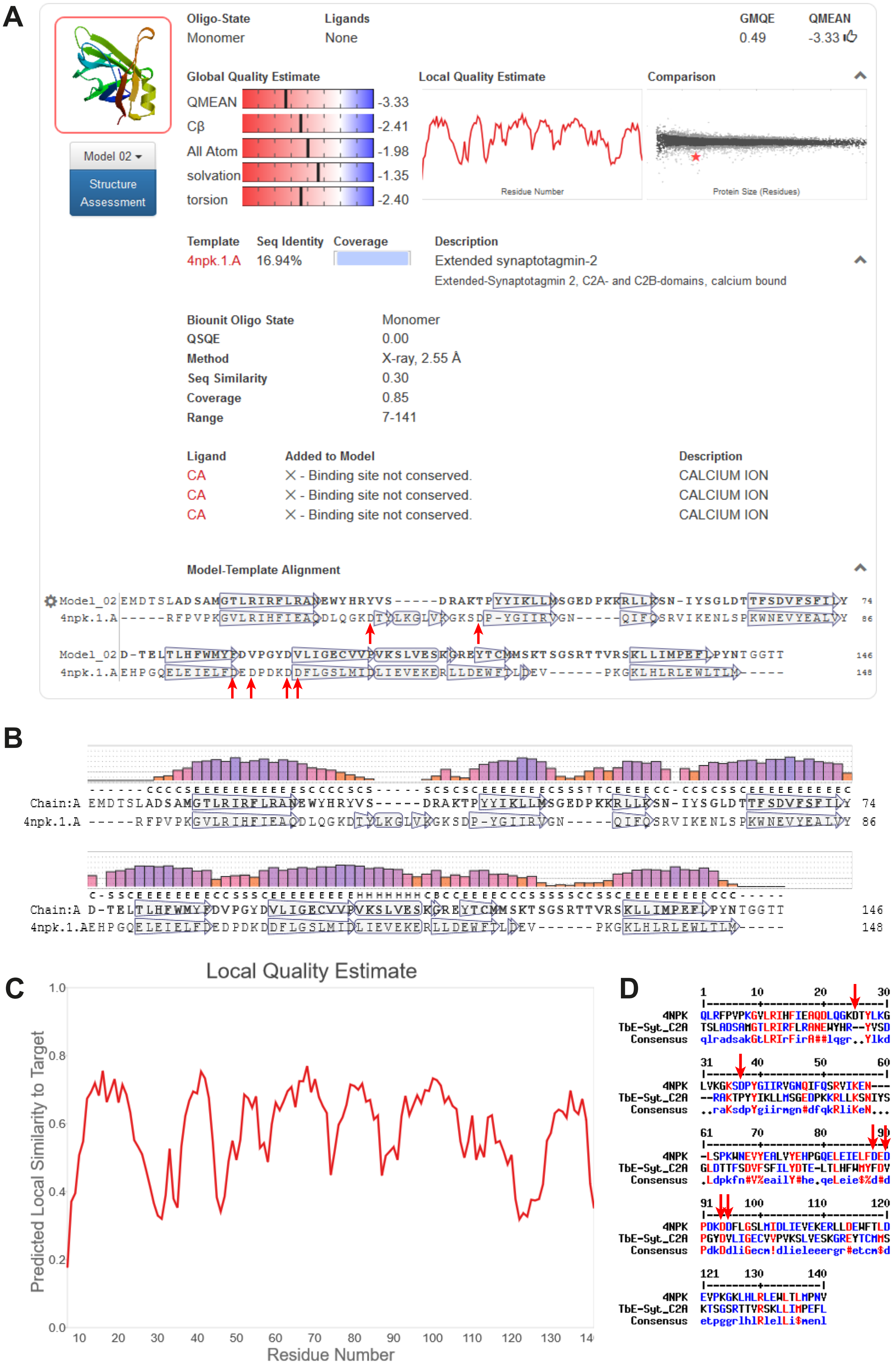
A
Oligo-State
Monomer
Ligands
None
GMQE
0.49
QMEAN
-3.33
Model 02
Structure Assessment
Global Quality Estimate
QMEAN -3.33
Cβ -2.41
All Atom -1.98
solvation -1.35
torsion -2.40
Local Quality Estimate
Residue Number
Comparison
Protein Size (Residues)
Template 4npk.1.A
Seq Identity 16.94%
Coverage
Description
Extended synaptotagmin-2
Extended-Synaptotagmin 2, C2A- and C2B-domains, calcium bound
Biounit Oligo State Monomer
QSQE 0.00
Method X-ray, 2.55 Å
Seq Similarity 0.30
Coverage 0.85
Range 7-141
Ligand
Added to Model
Description
CA ✕ - Binding site not conserved. CALCIUM ION
CA ✕ - Binding site not conserved. CALCIUM ION
CA ✕ - Binding site not conserved. CALCIUM ION
Model-Template Alignment
Model_02 EMDTSLADSAMGTLRIRFLRANEWYHRYVS-----DRAKTPYYIKLLMSGEDPKKRLLKSN-IYSGLDTTFSDVFSFILY 74
4npk.1.A -----RFPVPKGVLRIHFIEAQDLQGKDTYLKGLVKGKSDP-YGIIRVGN-----QIFQSRVIKENLSPKWNEVYEALVY 86
Model_02 D-TELTLHFWMYFDVPGYDVLIGECVVPVKSLVESKGREYTCMMSKTSGSRTTVRSKLLIMPEFLPYNTGGTT 146
4npk.1.A EHPGQELEIELFDEDPDKDDFLGSLMIDLIEVEKERLLDEWFTLDEV------PKGKLHLRLEWLTLMP----- 148
B
Chain:A EMDTSLADSAMGTLRIRFLRANEWYHRYVS-----DRAKTPYYIKLLMSGEDPKKRLLKSN-IYSGLDTTFSDVFSFILY 74
4npk.1.A -----RFPVPKGVLRIHFIEAQDLQGKDTYLKGLVKGKSDP-YGIIRVGN-----QIFQSRVIKENLSPKWNEVYEALVY 86
Chain:A D-TELTLHFWMYFDVPGYDVLIGECVVPVKSLVESKGREYTCMMSKTSGSRTTVRSKLLIMPEFLPYNTGGTT 146
4npk.1.A EHPGQELEIELFDEDPDKDDFLGSLMIDLIEVEKERLLDEWFTLDEV------PKGKLHLRLEWLTLMP----- 148
C
Local Quality Estimate
Predicted Local Similarity to Target
Residue Number
D
4NPK QLRFPVPKGVLRIHFIEAQDLQGKDTYLKG
TbE-Syt_C2A TSLADSAMGTLRIRFLRANEWYHR--YVSD
Consensus qlradsakGtLRIrFirA##lqgr..Ylkd
4NPK LVKGKSDPYGIIRVGNQIFQSRVIKEN---
TbE-Syt_C2A --RAKTPYYIKLLMSGEDPKKRLLKSNIYS
Consensus ..raKsdpYgiirmgn#dfqkRliKeN...
4NPK -LSPKWNEVYEALVYEHPGQELEIELFDED
TbE-Syt_C2A GLDTTFSDVFSFILYDTE-LTLHFWMYFDV
Consensus .Ldpkfn#V%eailY#he.qeLeie$%d#d
4NPK PDKDDFLGSLMIDLIEVEKERLLDEWFTLD
TbE-Syt_C2A PGYDVLIGECVVPVKSLVESKGREYTCMMS
Consensus PdkDdliGecm!dlieleeergr#etcm$d
4NPK EVPKGKLHLRLEWLTLMPNV
TbE-Syt_C2A KTSGSRTTVRSKLLIMPEFL
Consensus etpggrlhlRlelLi$menl

Figure S6. Homology modeling of TbE-Syt-C2A. Related to Figure 6. (**A**) Homology modeling of TbE-Syt-C2A by SWISS-MODEL using the HsE-Syt2-C2A structure (4NPK.pdb) as the template. Note that the three Ca^{2+}-binding sites, which are marked by arrows in the template, are not conserved in TbE-Syt-C2A. (**B**) Alignment of the TbE-Syt-C2A model with the template with secondary structures shown in the DSSP mode. (**C**) Local quality estimate for each residue in the final model. (**D**) Sequence alignment of HsE-Syt2-C2A (4NPK) and TbE-Syt-C2A using the hierarchical clustering based multiple sequence alignment tool MultAlin (http://multalin.toulouse.inra.fr/multalin/). Marked by arrows are the residues coordinating Ca^{2+} binding in HsE-Syt2-C2A. This further confirms that the absence of negatively residues at the putative Ca^{2+}-binding sites in TbE-Syt-C2A.

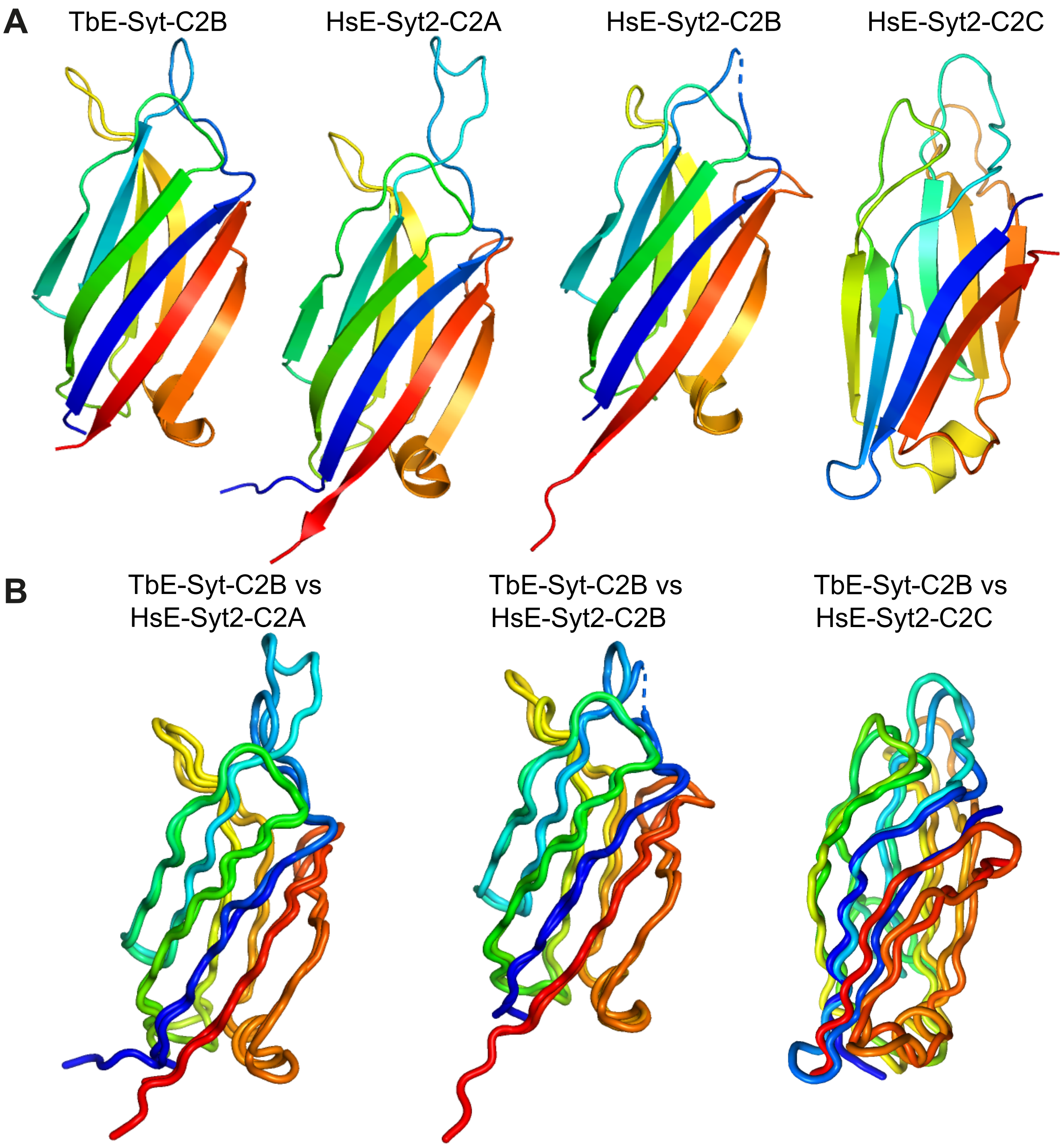

C

**TbE-Syt-C2B** vs **HsE-Syt2-C2A**<br>**1.05 Å**	**TbE-Syt-C2B** vs **HsE-Syt2-C2B**<br>**0.75 Å**	**TbE-Syt-C2B** vs **HsE-Syt2-C2C**<br>**3.57 Å**
	**HsE-Syt-C2A** vs **HsE-Syt2-C2B**<br>**0.99 Å**	**HsE-Syt-C2A** vs **HsE-Syt2-C2C**<br>**2.45 Å**
		**HsE-Syt-C2B** vs **HsE-Syt2-C2C**<br>**1.43 Å**

Figure S7. Structural comparison of TbE-Syt-C2B with the three C2 domains of HsE-Syt2. Related to Figure 7. (**A**) Ribbon diagrams of TbE-Syt-C2B and HsE-Syt2-C2A, C2B and C2C. All structures are rainbow colored from blue (N-terminus) to red (C-terminus). (**B**) TbE-Syt-C2B superimposed onto HsE-Syt2-C2A, C2B and C2C. (**C**) R.M.S.D values for each pair of superimposed structures.

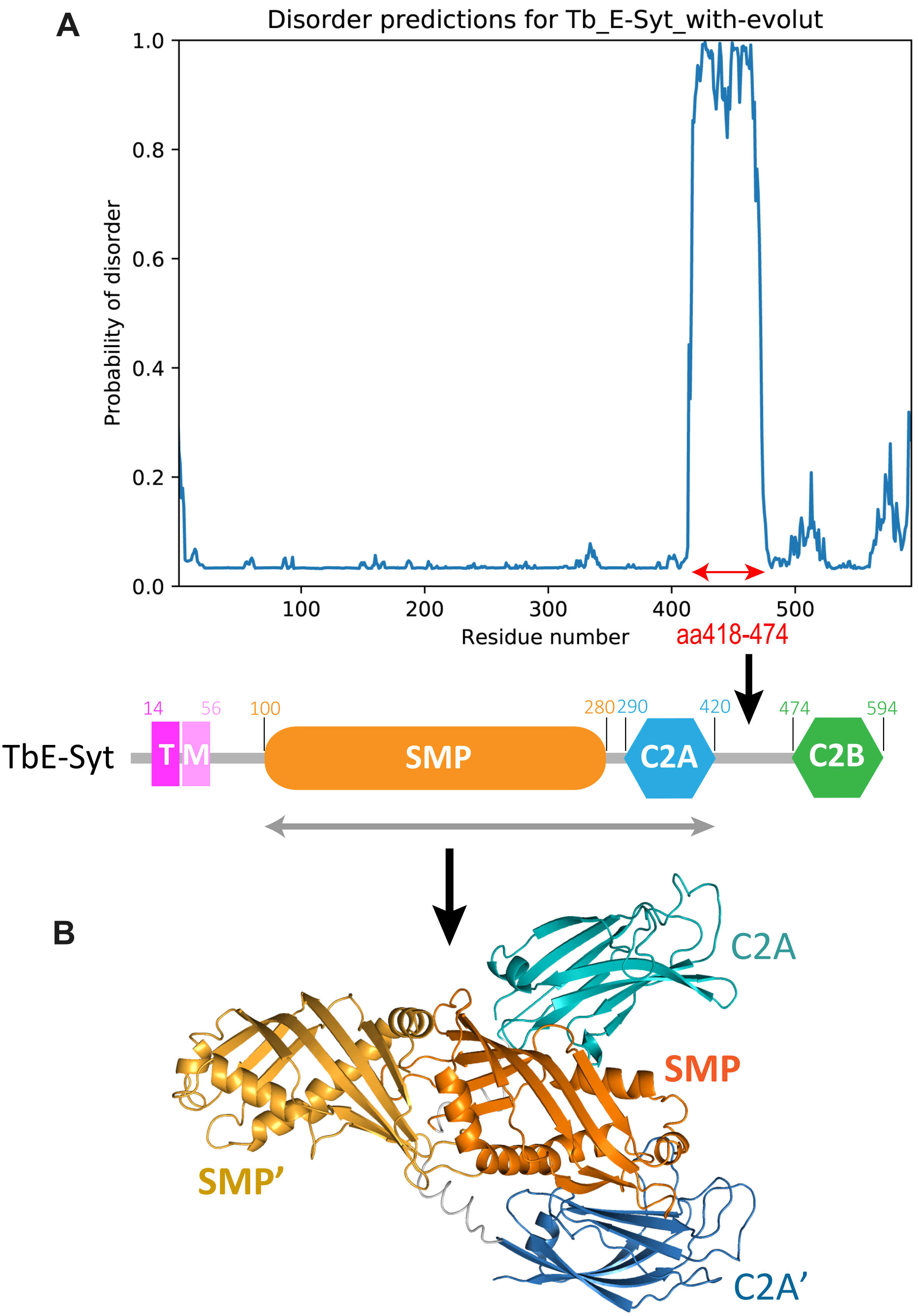

Figure S8. Folding and structure prediction of TbE-Syt. Related to Figure 7. (**A**) Disorder predictions of TbE-Syt by ODiNPred. Residues 418-474 of TbE-Syt was predicted to have very high probabilities of being disordered, which is consistent with the low conservation and variable lengths of this connecting loop in different homologs of TbE-Syt (see Fig. 1B). (**B**) Homology model of TbE-Syt-SMP-C2A (aa97-420) generated by SWISS-MODEL using the crystal structure of HsE-Syt2 (4NPK.pdb) as the template. The short linker (aa280-290) between the SMP and C2A domains, which are colored grey, suggests a relatively tight packing between these two domains. This is supported by predicted low disorder probability of this region (A) and is consistent with the structure of the counterpart in HsE-Syt2.

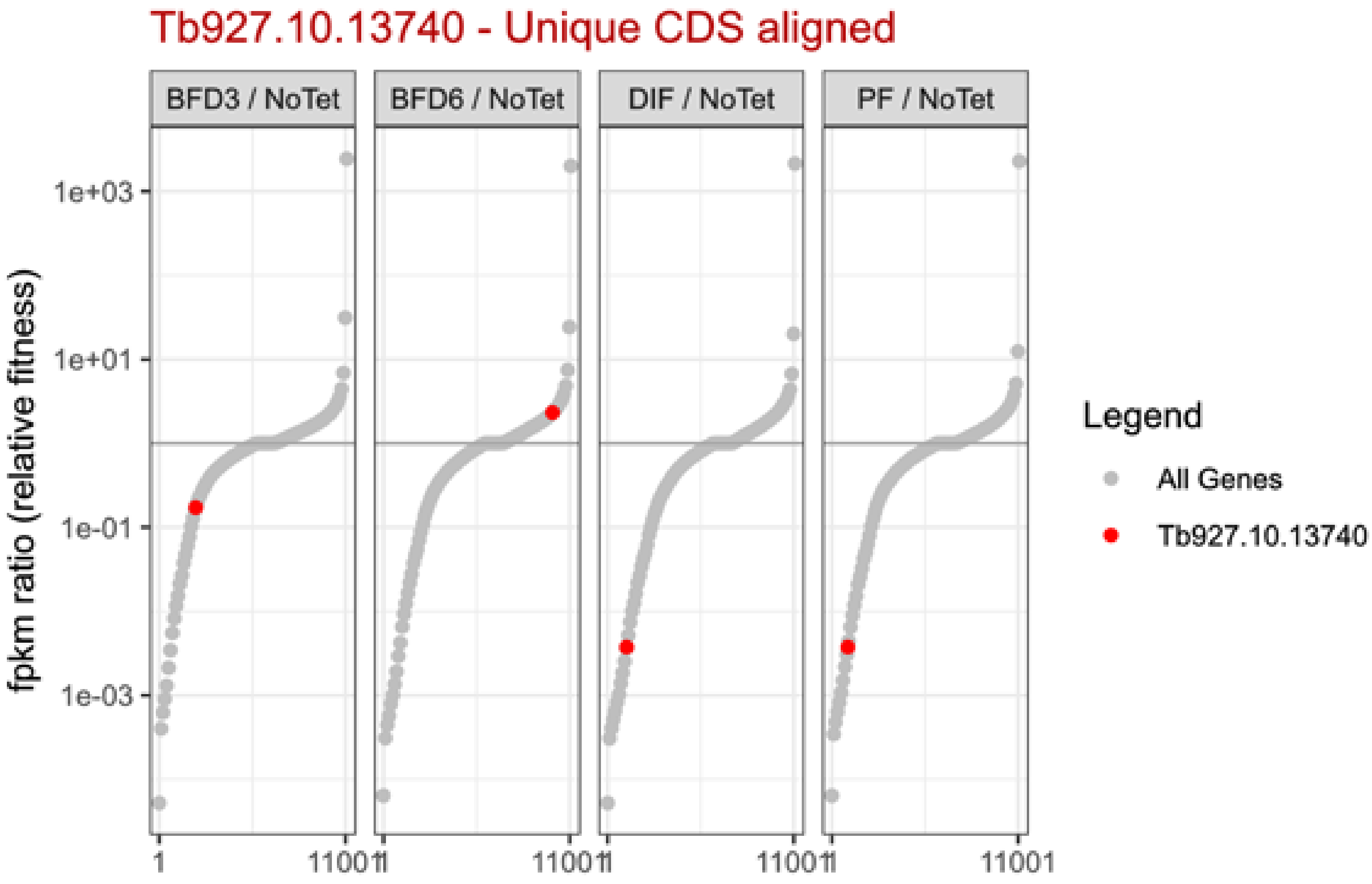

https://tritrypdb.org/tritrypdb/app/record/gene/Tb927.10.13740

Figure S9. Phenotypes of High-throughput TbE-Syt RNAi in *T. brucei* reported in the TriTrypDB database. Related to Figure 8. There was a very mild effect in the bloodstream form three (BFD3) and six days (BFD6) post RNAi induction. However, a strong negative score was recorded in the procyclic form, whether RNAi was induced in the procyclic form (PF) or during differentiation (DIF), which suggests that the protein is essential for cell viability of procyclic form *T. brucei*. Data copied with permission from TriTrypDB (http://tritrypdb.org).

Transparent Methods

Cloning and site-directed mutagenesis

For *in vivo* assays, the TbE-Syt ORF (Tb927.10.13740) was amplified by PCR from *T. brucei* TREU927/4 GUTat10.1 genomic DNA (Berriman et al., 2005) and cloned into modified pLew100 vector containing the puromycin resistance gene (Wirtz et al., 1999). This allowed tetracycline-inducible expression of 3xmyc C-terminal tagged TbE-Syt and various trucations (∆C2B: aa1-473; C2B: aa474-594; TM: aa1-96; ∆TM: aa97-594; C2A-SMP: aa97-473). The mutant E492A/D498A (mutC2B) was generated in pLew100-TbE-Syt-myc subsequently by site-directed mutagenesis using a QuikChange kit (Stratagene) according to the manufacturer's instructions. For the RNAi experiments in procyclic form and bloodstream form, a *TbE-Syt* fragment (bp 957-1581) was cloned into the double promoter p2T7^{TiB}-GFP vector (LaCount et al., 2002), and the pLew100 for stem-loop construct (Wirtz et al., 1999). All cloned constructs were verified by DNA sequencing.

For bacterial purification, full-length TbE-Syt (aa1-594) was amplified by PCR from the *T. brucei* genomic DNA and ligated into the expression vector pET15b (Novagen) between the *Nde*I and *BamH*I sites. Various truncations of TbE-Syt, including TM-SMP-C2A-C2B (aa60-594), TM-SMP-C2A (aa60-422), TM-SMP (aa60-285), SMP-C2A-C2B (aa94-594), SMP-C2A (aa94-422), SMP (aa94-285), C2A-C2B (aa282-594), C2A (aa282-422), and C2B (aa459-594), were subcloned from the full-length construct into both pET15b (Novagen) and a custom vector MalpET that provides a fusion tag of maltose-binding protein (MBP) to the N-terminus of the truncated fragments. However, except for the construct of C2B (aa459-594), none of the other truncations yielded soluble proteins.

TbE-Syt-C2B (aa459-594) cloned into pET15b yielded protein with an N-terminal His_6 tag that was cleavable by thrombin. The three mutants, E492A/D498A, K502E/K511E and E492A/D498A/K502E/K511E, were subsequently generated based on this construct by site-directed mutagenesis using the QuikChange kit (Stratagene) according to the manufacturer's instructions. Incorporations of mutations were confirmed by DNA sequencing.

Trypanosome cell lines, growth conditions and transfection

The TbE-Syt ORF (Tb927.10.13740) was amplified by PCR from genomic DNA of *T. brucei* TREU927/4 GUTat10.1 (Melville et al., 2000). The trypanosome cell lines *T. brucei* 427 procyclic form 29-13 and bloodstream form 90-13 co-expressing the T7 RNA polymerase and tetracycline repressor (Wirtz et al., 1999) were grown and transfected as described before (Pradel et al., 2006). After transfection, the cells were selected with puromycin (1 µg/ml) and phleomycin 5 µg/ml (procyclic form) and 2.5 µg/ml (bloodstream form) as appropriate. Clones were selected after serial dilutions. Ectopic expression and

RNAi were induced with tetracycline at 10 ng/ml (except TbE-Syt $TM_{::myc}$ expression that was induced at 100 ng/ml) and 1-10 μg/ml, respectively.

Protein expression and purification

Recombinant His_6-TbE-Syt-C2B and the three mutants were expressed in the *E. coli* strain BL21(DE3). The bacterial cells transformed with individual constructs were grown in Luria-Bertani (LB) medium at 37°C with continuous agitation to an OD_{600} of 0.6-0.8, and then placed on ice for 30 min (cold shock). Induction of protein expression was done by addition of 0.5 mM isopropyl β-D-1-thiogalactopyranoside (IPTG), and the cells were further incubated at 16°C overnight (14-16 h) with continuous agitation. Bacterial cells were harvested by centrifugation in a Sorvall GS3 rotor (6,000×g, 12 min, 4°C), and the pellet was resuspended in 20 ml of lysis buffer (20 mM Tris-HCl pH 8.0, 100 mM NaCl, 20 mM imidazole, 5% (v/v) glycerol) per liter of cell culture.

Harvested cells were lysed in an EmulsiFlex-C3 homogenizer (Avestin) and cell debris was removed by centrifugation (40,000×g, 40 min, 4°C). The resulting supernatant was filtered (0.45-μm, Amicon) and loaded onto a 5-ml Ni-HiTrap column (GE Healthcare) that was pre-equilibrated with the same lysis buffer. The column was washed with 5 × column volume (cv) of lysis buffer, and bound protein was subsequently eluted using a linear gradient concentration of imidazole (20 - 500 mM, 20×cv) in the same lysis buffer.

The N-terminal His_6 tag was removed by incubating the pooled fractions containing wild-type or mutant TbE-Syt-C2B with ~2% (w/w) of thrombin (4°C, overnight). Target proteins were further purified on a Superdex-200 16/60 column (GE Healthcare), which was pre-equilibrated with buffer containing 20 mM Tris-HCl (pH 8.0) and 100 mM NaCl. The eluted proteins were pooled and concentrated using the centrifugal filters (MWCO 3,000, Amicon), and protein concentration was determined using ultraviolet (UV) absorbance at 280 nm and double-checked on SDS-PAGE gels for quality control.

Se-Met-substituted TbE-Syt-C2B was expressed following previously reported protocols (Doublie, 1997). Purification was carried out using the same protocol as for the wild-type protein, except that 15 mM β-mercaptoethanol and 10 mM DTT was added to the lysis buffer and the gel filtration buffer, respectively. The purified protein was concentrated to ~15 mg/ml for crystallization trials.

Crystallization, data collection and structure determination

Small clustered crystals of TbE-Syt-C2B were originally obtained in a large-scale crystallization screening trial using native protein and commercial crystallization screening kits (Hampton Research). Rod-shaped crystals of Se-Met-substituted TbE-Syt-C2B were subsequently obtained upon extensive optimization tests. The final crystallization conditions contained 0.1 M sodium cacodylate (pH 6.5), 0.15 M sodium acetate, and 28%

(v/v) polyethylene glycol 8,000. Crystals were harvested by sequentially soaking in the same reservoir solution with 5%, 10% and 15% (v/v) glycerol, loop mounted, and then flash frozen in liquid nitrogen. Diffraction data were collected at the beamline ID23-2 of the European Synchrotron Radiation Facility (ESRF), which ran at the fixed wavelength of 0.873 Å (14.2 keV). To minimize radiation damage, the data used for final structure determination were collected using the helical data acquisition mode to scan along the long axis of the crystal rod. All data were integrated and scaled using XDS (Kabsch, 2010).

Structure determination was carried out using the single-wavelength anomalous diffraction method. Selenium sites were located and experimental electron density maps calculated using the program AutoSol within the Phenix suite (Terwilliger et al., 2009). Partially built models were checked and missing loops were manually added in COOT (Emsley and Cowtan, 2004). Refinement was done using phenix.refine (Afonine et al., 2012).

Liposome pelleting assay with sucrose-loaded vesicles

Sucrose-loaded vesicles were prepared as follows: all lipids were reconstituted in chloroform and mixed in molar ratio of 20% cholesterol, 15% DOPS, 35% DOPE, and 30% DOPC, with 60 µM rhodamine dye. PI(4,5)P_2-containing vesicles had 5% of PI(4,5)P_2 and 25% of DOPC. The lipid mixture was dried to a thin film under weak nitrogen stream, hydrated in 20 mM HEPES (pH 7.5), 0.3 M sucrose and vortexed vigorously for 1 min at RT. The multilamellar vesicles created by this process were then subjected to four cycles of freezing in liquid nitrogen followed by thawing in a sonicating water bath at RT. This mixture of small unilamellar vesicles was diluted with 20 mM HEPES (pH 7.5), 100 mM KCl and pelleted at 230,000 × g for 30 min at RT. The pelleted vesicles were resuspended in 20 mM HEPES (pH 7.5), 100 mM KCl and incubated with purified protein at a 1:1 ratio for 30 min at RT. For Ca^{2+}-mediated liposome binding tests, proteins were incubated with 2 mM $CaCl_2$ for 5 min at RT prior to mixing with the vesicles. To separate soluble and sucrose-loaded vesicles-bound proteins, the mixtures were pelleted at 8000 × g for 30 min at RT, and equal volumes of supernatant and resuspended pellets were analyzed on SDS-PAGE gels.

Western blotting

Cells (2×10^7) were split for whole cells (WC) and cytoskeleton (CSK) samples and processed for western blotting analysis as described before (Florimond et al., 2015). Briefly, protein samples (equivalent to 5×10^6 trypanosomes whole cell or cytoskeletons) were separated on 15% (w/v) SDS-PAGE gels and transferred by semi-dry (BioRad) blotting to PVDF membranes. After blocking, the membranes were incubated overnight at 4°C with the anti-myc (9E10 mouse monoclonal, 1:10,000) primary antibody. After washes in 1 M NaCl and in blocking solution, the membranes were incubated for 1 h at RT with

the secondary antibody [anti-mouse HRP-conjugated (Jackson, 1:10,000)]. Blots were visualized using the Clarity Western ECL Substrate kit (Bio-Rad) on an ImageQuant LAS4000. After stripping in 100 mM glycine (pH 2.3), 0.1% (v/v) IGEPAL CA-630 and 1% (w/v) SDS, the membranes were processed as above with anti-TbSAXO (mAb25) antibodies as a loading control (Dacheux et al., 2012).

Immunofluorescence and microscopy

Wide-field fluorescence microscopy on *T*. *brucei* cells were processed for whole cell (WC) or detergent-extracted cytoskeletons (CSK) immunolabeling as described before (Florimond et al., 2015). Briefly, the slides were incubated with primary antibodies [anti-BiP (Bangs et al., 1993) 1:4,000, anti-myc (monoclonal 9E10) 1:20, L8C4 neat, and L3B2 1:25] and washed 2 x 5 min in PBS, then incubated with the secondary antibodies [anti-rabbit Alexa594-conjugated (Fischer A11012) 1:100; anti-mouse FITC-conjugated (Sigma F-2012)]. After DAPI staining (5 min, 10 µg/ml in PBS), the slides were mounted with SlowFAde Gold Antifade reagent. Images were acquired on a Zeiss Imager Z1 microscope with a Zeiss 100x objective (NA 1.4), using a Photometrics Coolsnap HQ2 camera and Metamorph software (Molecular Devices), and processed with ImageJ. Pearson's correlation coefficients were calculated (n = 22) for the TbE-Syst constructs FL, T1, T3, and mutC2B using the imageJ JaCoP plugin (Bolte and Cordelieres, 2006) and Costes's randomization (Costes et al., 2004). The large cytosolic pool of T4 and T2 prevented the analysis.

Immuno-cryoEM was performed by Dr. Wandy Beatty (Washington University) as described previously (Zhou et al., 2011). Briefly, cells stably expressing TbE-Syt-YFP were fixed with 4% (w/v) paraformaldehyde solution and 0.2% (w/v) glutaraldehyde (EMS) in 100 mM phosphate buffer (pH 7.2) for 1 h at 4°C. The fixed cells were embedded in 10% gelatin and infiltrated with 2.3 M sucrose, 20% polyvinyl pyrrolidone in PIPES-NaOH (pH 6.9) containing 1 mM $MgCl_2$. Liquid nitrogen frozen sections were obtained using a Leica Ultracut UCT cryoultramicrotome (Leica Microsystems, Bannockburn, IL), and processed for immunolabeling with anti-GFP antibodies (Abcam, UK), followed with secondary antibodies conjugated to colloidal gold (Jackson ImmunoResearch Laboratories). Labeled sections were visualized with a JEOL 1200 EX electron microscope (JEOL).

Supplemental References

Afonine, P.V., Grosse-Kunstleve, R.W., Echols, N., Headd, J.J., Moriarty, N.W., Mustyakimov, M., Terwilliger, T.C., Urzhumtsev, A., Zwart, P.H., and Adams, P.D. (2012). Towards automated crystallographic structure refinement with phenix.refine. Acta Crystallogr D Biol Crystallogr *68*, 352-367.

Bangs, J.D., Uyetake, L., Brickman, M.J., Balber, A.E., and Boothroyd, J.C. (1993). Molecular cloning and cellular localization of a BiP homologue in Trypanosoma brucei. Divergent ER retention signals in a lower eukaryote. J Cell Sci *105 (Pt 4)*, 1101-1113.

Berriman, M., Ghedin, E., Hertz-Fowler, C., Blandin, G., Renauld, H., Bartholomeu, D.C., Lennard, N.J., Caler, E., Hamlin, N.E., Haas, B.*, et al.* (2005). The genome of the African trypanosome Trypanosoma brucei. Science *309*, 416-422.

Bolte, S., and Cordelieres, F.P. (2006). A guided tour into subcellular colocalization analysis in light microscopy. J Microsc *224*, 213-232.

Costes, S.V., Daelemans, D., Cho, E.H., Dobbin, Z., Pavlakis, G., and Lockett, S. (2004). Automatic and quantitative measurement of protein-protein colocalization in live cells. Biophys J *86*, 3993-4003.

Dacheux, D., Landrein, N., Thonnus, M., Gilbert, G., Sahin, A., Wodrich, H., Robinson, D.R., and Bonhivers, M. (2012). A MAP6-related protein is present in protozoa and is involved in flagellum motility. PLoS One *7*, e31344.

Doublie, S. (1997). Preparation of selenomethionyl proteins for phase determination. Methods Enzymol *276*, 523-530.

Emsley, P., and Cowtan, K. (2004). Coot: model-building tools for molecular graphics. Acta Crystallogr D Biol Crystallogr *60*, 2126-2132.

Florimond, C., Sahin, A., Vidilaseris, K., Dong, G., Landrein, N., Dacheux, D., Albisetti, A., Byard, E.H., Bonhivers, M., and Robinson, D.R. (2015). BILBO1 is a scaffold protein of the flagellar pocket collar in the pathogen Trypanosoma brucei. PLoS Pathog *11*, e1004654.

Kabsch, W. (2010). Xds. Acta Crystallogr D Biol Crystallogr *66*, 125-132.

LaCount, D.J., Barrett, B., and Donelson, J.E. (2002). Trypanosoma brucei FLA1 is required for flagellum attachment and cytokinesis. J Biol Chem *277*, 17580-17588.

Melville, S.E., Leech, V., Navarro, M., and Cross, G.A. (2000). The molecular karyotype of the megabase chromosomes of Trypanosoma brucei stock 427. Mol Biochem Parasitol *111*, 261-273.

Pradel, L.C., Bonhivers, M., Landrein, N., and Robinson, D.R. (2006). NIMA-related kinase TbNRKC is involved in basal body separation in Trypanosoma brucei. J Cell Sci *119*, 1852-1863.

Terwilliger, T.C., Adams, P.D., Read, R.J., McCoy, A.J., Moriarty, N.W., Grosse-Kunstleve, R.W., Afonine, P.V., Zwart, P.H., and Hung, L.W. (2009). Decision-making in structure solution using Bayesian estimates of map quality: the PHENIX AutoSol wizard. Acta Crystallogr D Biol Crystallogr *65*, 582-601.

Wirtz, E., Leal, S., Ochatt, C., and Cross, G.A. (1999). A tightly regulated inducible expression system for conditional gene knock-outs and dominant-negative genetics in Trypanosoma brucei. Mol Biochem Parasitol *99*, 89-101.

Zhou, Q., Liu, B., Sun, Y., and He, C.Y. (2011). A coiled-coil- and C2-domain-containing protein is required for FAZ assembly and cell morphology in Trypanosoma brucei. J Cell Sci *124*, 3848-3858.

KEY RESOURCES TABLE

REAGENT or RESOURCE	SOURCE	IDENTIFIER
**Antibodies**		
Anti-GFP	Abcam	ab6556
Anti-PFR	Zhang et al., 2018	https://jcs.biologists.org/content/131/17/jcs219071
L3B2	Kohl et al., 1999	https://onlinelibrary.wiley.com/doi/abs/10.1111/j.1550-7408.1999.tb04592.x
Anti myc clone 9E10	K. Ersfeld	N/A
Anti BiP	Bangs et al., 1993	https://jcs.biologists.org/content/105/4/1101
Anti-TbSAXO	Dacheux et al., 2012	https://doi.org/10.1371/journal.pone.0031344"
Anti-PFR L8C4	Kohl et al., 1999	https://doi.org/10.1111/j.1550-7408.1999.tb04592.x
**Bacterial and virus strains**		
E. coli BL21(DE3)	Novagen	Cat#69450
E. coli HST08 (stellar)	Clontech	Cat#636766
Biological samples		
**Chemicals, peptides, and recombinant proteins**		
TbE-Syt-C2B (aa459-594)	This paper	N/A
**Critical commercial assays**		
Deposited data		
TbE-Syt-C2B structure	This paper	PDB: 7A1R

**Experimental models: cell lines**		
T. brucei TREU927/4 GUTat10.1	Berriman et al., 2005	https://science.sciencemag.org/content/309/5733/416.long
T. brucei 427 29.13	Wirtz et al., 1999	https://science.sciencemag.org/content/268/5214/1179
**Experimental models: organisms/strains**		
**Oligonucleotides**		
Primer: TbE-Syt-C2B Forward: GCAGCTGCATATGAGCACTCGCTCGACGGTGCC	This paper	N/A
Primer: TbE-Syt-C2B Reverse: CGACGGATCCTTAGTGGCGCAGAAGCTTAAG	This paper	N/A
Primer : TbE-Syt-ORF, TbE-Syt-ΔC2B, TbE-Syt-TM Forward : AAAATTCACaagcttATGGAAAAGGCAAATGAACTGATGCA	This Paper	N/A
Primer : TbE-Syt-ORF, TbE-Syt-C2B, TbE-Syt-ΔTM Reverse : CCCGGTACCGGATCCgtggcgcagaagcttaagttcaag	This Paper	N/A
Primer : TbE-Syt-ΔC2B, TbE-Syt-C2A-SMP Reverse : CCCGGTACCggatccgtggttttcaacaccatcatcgttc	This Paper	N/A
Primer : TbE-Syt-C2B Forward : AAAATTCACaagcttatggggggtggaacgcttttcgtc	This Paper	N/A
Primer : TbE-Syt-TM Forward : CCCGGTACCggatccgctggggttgatgagccactc	This Paper	N/A
Primer : TbE-Syt-ΔTM, TbE-Syt-C2A-SMP Forward : AAAATTCACaagcttatggcgaacaatgtccagtggttgaac	This Paper	N/A
Primer : TbE-Syt-mutC2B E492A Forward : ctgaagaacaaagCaactattggggtctc	This Paper	N/A
Primer : TbE-Syt-mutC2B E492A Reverse : GAGACCCCAATAGTTGCTTTGTTCTTCAG	This Paper	N/A
Primer : TbE-Syt-mutC2B D498A Forward : ctattggggtctccgCcccatacgtcaagttac	This Paper	N/A
Primer : TbE-Syt-mutC2B D498A Reverse : GTAACTTGACGTATGGGGCGGAGACCCCAATAG	This Paper	N/A
Primer : TbE-Syt RNAi Stem-loop sense Forward : TGTAAGCTTCAAACTGTTGATGTCCGGTGA	This Paper	N/A
Primer : TbE-Syt RNAi Stem-loop sense Reverse : TGTCTCGAGACTTGACGTATGGGTCGGAGA	This Paper	N/A
Primer : TbE-Syt RNAi Stem-loop antisense and RNAi double promoter Forward : TGTGGATCCCAAACTGTTGATGTCCGGTGA	This Paper	N/A

**Recombinant DNA**		
Plasmid: pLew100	Wirtz et al., 1999	https://www.sciencedirect.com/science/article/pii/S016668519900002X?via%3Dihub
Plasmid: p2T7TiB-GFP	LaCount et al., 2002	https://www.jbc.org/article/S0021-9258(20)85247-8/fulltext
Plasmid: pET15b	Novagen	Cat. No. 69661-3
Plasmid: MalpET	This paper	N/A
**Software and algorithms**		
COOT	Emsley, et a., 2004	http://scripts.iucr.org/cgi-bin/paper?S0907444904019158
XDS	Kabsch, 2010	https://onlinelibrary.wiley.com/iucr/doi/10.1107/S0907444909047337
PHENIX AutoSol	Terwilliger, et al., 2009	http://scripts.iucr.org/cgi-bin/paper?S0907444909012098
phenix.refine	Afonine, et al., 2012	http://scripts.iucr.org/cgi-bin/paper?S0907444912001308
ImageJ	Schneider et al., 2012	https://imagej.nih.gov/ij/
JACoP (ImageJ plugin)	Bolte et al., 2006	https://imagej.net/JaCoP
Costes's randomization	Costes et al., 2004	https://www.sciencedirect.com/science/article/pii/S0006349504744392?via%3Dihub
Other		
